# Halogen bonding-guided growth of heteroatom-rich polycarbazole wires on Au(111)

**DOI:** 10.1039/d5na00708a

**Published:** 2025-08-20

**Authors:** Frank Palmino, Vincent Luzet, Judicaël Jeannoutot, Alain Rochefort, Frédéric Chérioux

**Affiliations:** a Université Marie et Louis PASTEUR, FEMTO-ST, CNRS F-25000 Besançon France frederic.cherioux@femto-st.fr; b Département de génie physique, Polytechnique Montréal Montréal (Québec) – H3C 3A7 Canada

## Abstract

The growth of conjugated polymers has been widely investigated on metal surfaces in ultrahigh vacuum. Here, we report that pre-organized halogen-bonded templates, based on a selecting bent molecular geometry, enable the on-surface synthesis of long, defect-free polycarbazole wires, laterally decorated with nitrogen-bound substituents as revealed by scanning tunnelling microscopy.

## Abstract 2

The growth of long, defect-free polycarbazole wires is achieved by using a pre-organized halogen-bonded templates, based on a selecting bent molecular geometry.

## Graphical abstract

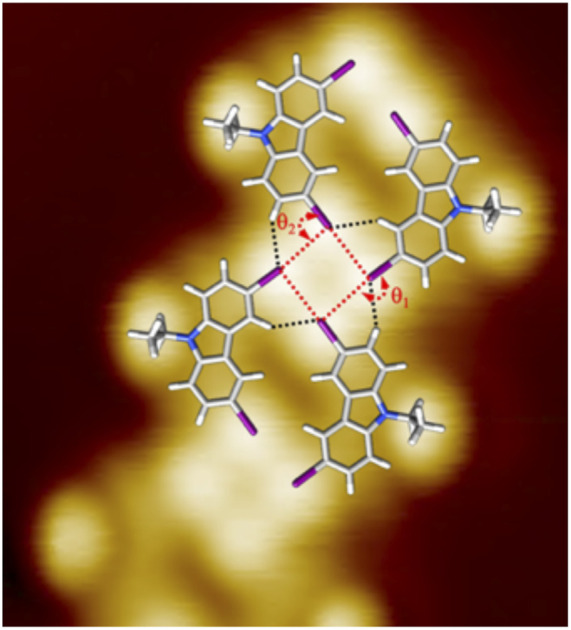

Conjugated polymers have garnered significant interest due to their unique electronic, optical, and mechanical properties, which make them suitable candidates for applications in organic electronics, optoelectronics, sensors, and functional nanomaterials. When confined to surfaces-particularly metallic substrates-these materials can self-assemble into low-dimensional architectures with tunable properties, offering pathways toward bottom-up fabrication of molecular-scale devices.^1–3^ On-surface polymerization under ultra-high vacuum (UHV) has emerged as a powerful strategy to fabricate atomically precise polymeric nanostructures directly on surfaces.^4–6^ This approach can be enhanced by pre-organizing the molecular precursors to facilitate the formation of covalent bonds between monomers, thereby minimizing defects during polymer growth. Among all types of supramolecular interactions, halogen bonds are particularly effective due to their strong directionality and their role in the creation of complex C–C networks *via* dehalogenation such as graphynes.^7–15^ We have recently demonstrated that the use of X_3_-synthons enables the growth of one-dimensional polymers, even when the initial supramolecular organization is two-dimensional.^15^ In addition, surface-sensitive techniques such as UHV scanning tunneling microscopy (STM) offer direct visualization of the polymer structure, conformation, and substrate registry with sub-nanometer resolution.^16^ Heteroatom-containing conjugated polymers represent a particularly valuable subclass, as the presence of atoms such as nitrogen, oxygen, or sulfur introduces electronic anisotropy, modulates the charge distribution, and enables site-specific interactions, all of which can be leveraged to tailor the polymer's optoelectronic response.^17,18^ Carbazole derivatives, which feature a nitrogen heteroatom embedded in a rigid, planar π-conjugated system, are widely used in organic electronics owing to their thermal stability, high hole mobility, and efficient charge transport.^19,20^ Despite their potential, the surface-assisted polymerization of carbazole-based building blocks and their *in situ* characterization at the atomic scale remain underexplored.^21–24^ In this work, we report the formation and structural characterization of polycarbazole chains on Au(111) *via* thermally induced on-surface polymerization of a designed molecular precursor under UHV conditions. On the Au(111) surface, the polymerization of halogenated molecules is governed by thermodynamic control. The radicals produced *via* homolytic cleavage of C–halogen bonds exhibit notably long lifetimes, which opens the possibility for controlled covalent network formation.^25^ In this context, we investigate whether an initially formed one-dimensional (1D) supramolecular structure can guide the formation of an ordered covalent network upon thermal activation, by facilitating the diffusion of iodinated carbazole derivatives on the Au(111) surface. To explore this, we synthesized and then deposited an iodinated carbazole molecule (3,6-diiodo-9-ethylcarbazole –DIDEC–) at low temperatures and examined the effects of subsequent thermal annealing using UHV-STM. Our results show that at low temperatures, the supramolecular assembly is stabilized through quadruple halogen bonding. Moreover, the geometry of this supramolecular arrangement favors the formation of well-ordered 1D covalent networks upon annealing above 400 K. STM imaging, as well as simulated STM images by using density functional theory (DFT) calculations, reveal extended, ordered polymer chains, enabling detailed analysis of their spatial organization and bonding motifs. This study provides a model system for investigating the structure–property relationships of heteroatom-rich conjugated polymers on surfaces and opens avenues for the design of molecular electronic architectures with tailored functionalities.

We selected the Au(111) surface as it enables the Ullmann coupling reaction while promoting the diffusion of intermediate species.^25^ We chose the 3,6-diiodo-9-ethylcarbazole molecule (Fig. 1a) because the iodine atoms promote the formation of supramolecular structures on various types of surfaces and can be thermally activated to form polymers. The synthesis of molecules and surface preparation are fully described in electronic supplementary information (Scheme S1, Fig. S1 and 2). The DIDEC molecules were deposited by thermal sublimation under UHV on a Au(111) substrate maintained at a temperature below 80 K to avoid deiodination.

**Fig. 1:**
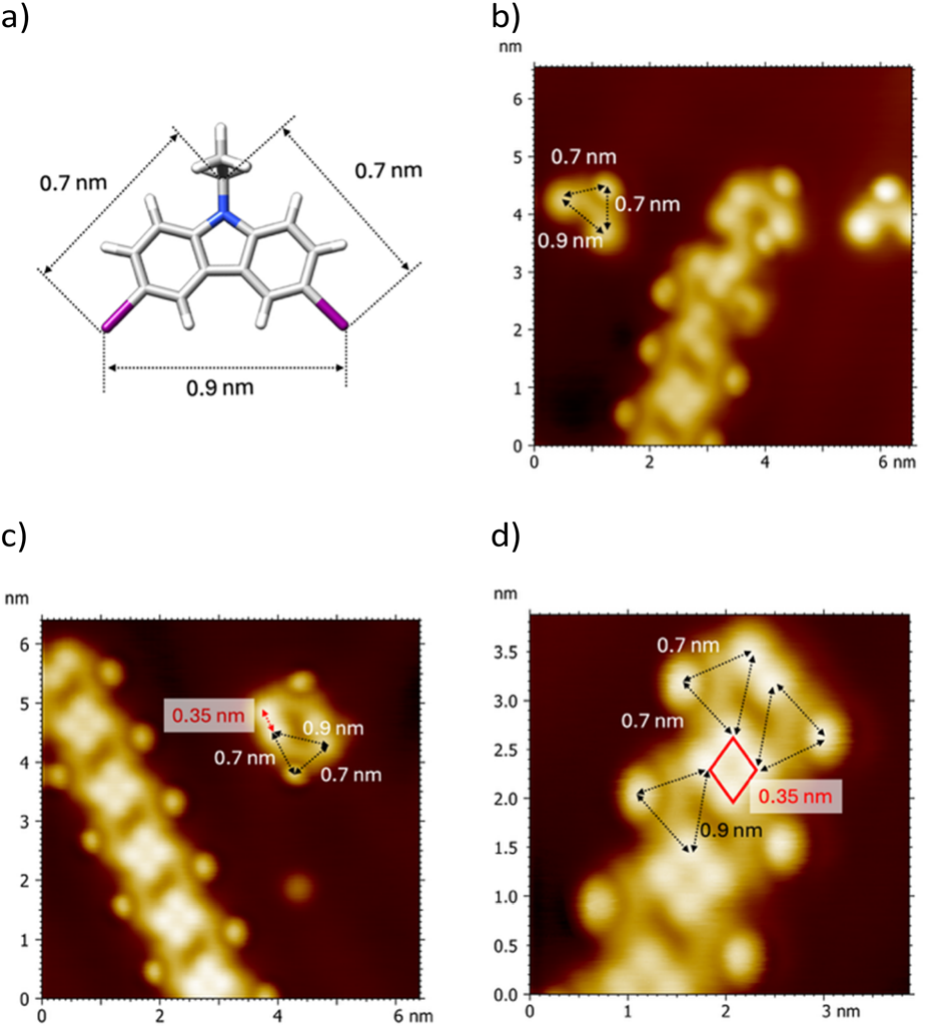
Different nanostructures observed after the deposition of 0.3 monolayer of DIDEC molecule onto an Au(111) surface at 80 K. (a) CPK model of the 3,6-diiodo-9-ethylcarbazole (DIDEC) molecule. (b) STM image (6.5 × 6.5 nm^2^, *V*_s_ = 0.3 V, *I*_t_ = 12 pA, 9 K) showing a bean-shaped protrusion surrounded by three bright spots arranged in an isosceles triangular geometry, with two sides measuring 0.7 nm and one side 0.9 nm. (c) STM image (6.4 × 6.4 nm^2^, *V*_s_ = 0.2 V, *I*_t_ = 180 pA, 9 K) showing two bean-shaped protrusions arranged face-to-face along the longest side, separated by 0.35 nm. The four closely spaced protrusions appear brighter than the two more distant ones. (d) STM image (3.9 × 3.9 nm^2^, *V*_s_ = −1.0 V, *I*_t_ = 31 pA, 9 K) showing a row composed of the features observed in (c). The four brightest protrusions are arranged in a square-shaped configuration, with sides measuring 0.35 nm.

STM images obtained at 9 K after the deposition of DIDEC molecules reveal the formation of various nanostructures (Fig. S3). Occasionally, isolated bean-shaped protrusions surrounded by three bright spots arranged in an isosceles triangular geometry—with two sides of 0.70 ± 0.05 nm and one of 0.90 ± 0.05 nm—are observed (Fig. 1b). These individual features also appear as dimers, arranged face-to-face along their longest sides, separated by 0.35 ± 0.05 nm (Fig. 1c). The most frequently observed motif, however, is a linear row consisting of a one-dimensional arrangement of these nanostructures. Each row is formed by repeating units of four isosceles triangles sharing two vertices, resulting in a diamond-shaped motif (highlighted in red in Fig. 1d) with an edge length of 0.35 nm. Within these rows, the apices of the isosceles triangles consistently point outward.

Based on their size and symmetry, the triangular features observed in STM images (Fig. 1b) are assigned to individual pristine DIDEC molecules. A proposed adsorption model is shown in Fig. 2a. Accordingly, the observed dimer (Fig. 1c) consists of two DIDEC molecules (see superimposed model in Fig. 2c). The angle between the two iodine atoms (denoted *θ*_1_ in Fig. 2c) is 180°, while the angle between the two C–I bonds (*θ*_2_) is 90°. The distance between the two iodine atoms is 0.35 ± 0.05 nm (black dashed in Fig. 2c), consistent with type II halogen–halogen interactions.^7^ Extending this analysis, each unit cell within the row shown in Fig. 1d is composed of four DIDEC molecules (model in Fig. 2e). The diamond shape corresponds to a square of four iodine atoms, each separated by 0.35 ± 0.05 nm (red dashed line in Fig. 2e). The angles *θ*_1_ and *θ*_2_ remain 180° and 90°, respectively. In the proposed model, one hydrogen atom from each DIDEC molecule points toward an adjacent iodine atom at 0.30 nm (black dashed lines in Fig. 2e). This arrangement corresponds to an X_4_-synthon involving four type II halogen–halogen interactions and four hydrogen–halogen interactions.^7^ The assignment of the observed protrusions is further supported by DFT and STM simulations (see ESI for a full description of methods), which provide insights into the chemical nature and composition of the frontier orbitals of the adsorbed species. Based on simulated STM images, the brightest features in the experimental STM images are attributed to the iodine atoms and the ethyl groups of the DIDEC molecules. In contrast, the bean-shaped protrusions are assigned to the aromatic backbone of the DIDEC molecules (Fig. 2b, d, and f). The cohesion energies of the DIDEC X_4_-synthon compatible with a 1D and 2D structure were evaluated using DFT calculations. The resulting values are −0.32 eV and −0.48 eV per DIDEC molecule for the 2D and the 1D X_4_-synthon, respectively (see Fig. S4). These interactions are significantly stronger than previously reported values for similar halogen-bonded assemblies, typically around −0.20 eV per molecule.^26^ This improving cohesion are related to additional intermolecular interactions in 1D and the use of a finite molecular model to mimic the assemblies. Notably, the cohesion energy of the 1D X_4_-synthon is approximately 50% higher than that of the 2D structure, which is consistent with the predominant observation of the X_4_-synthon motif over a 2D network in the STM images.

**Fig. 2:**
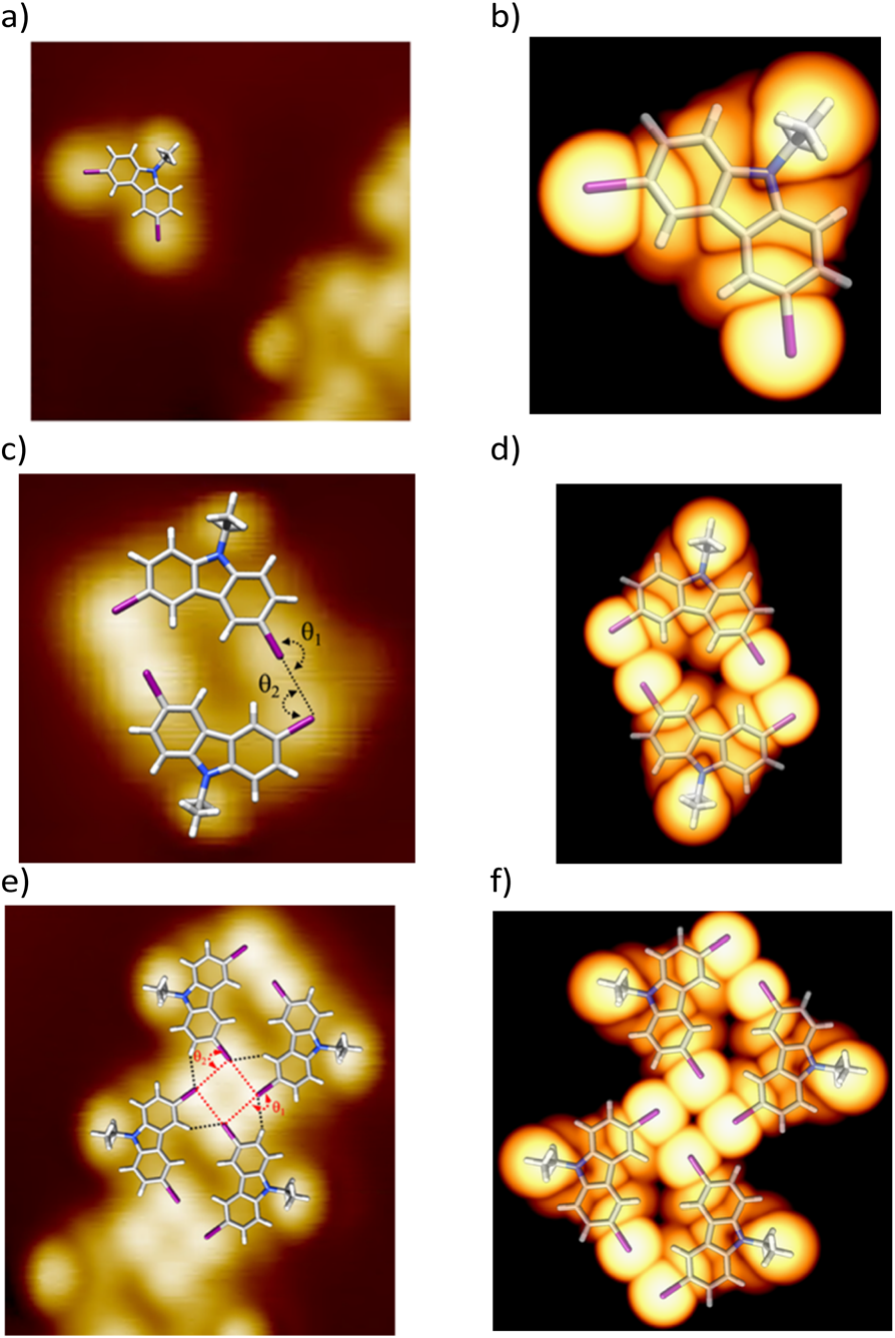
STM analysis of nanostructures formed upon deposition of 0.3 monolayers of DIDEC on Au(111) at 80 K. (a) Molecular model of DIDEC overlaid on the STM image from Fig. 1b; isosceles triangular features correspond to individual DIDEC molecules. (c) Molecular model overlaid on the STM image from Fig. 1c; two iodine atoms are separated by 0.35 nm. The angles *θ*_1_ = 180° and *θ*_2_ = 90° indicate a type-II halogen bond between DIDEC molecules (highlighted by black dashed line). (e) Molecular model overlaid on the STM image from Fig. 1d; iodine atoms are 0.35 nm apart with *θ*_1_ ≈ 180° and *θ*_2_ = 90°, characteristic of an X_4_-synthon formed by four DIDEC molecules (highlighted by black and red dashed lines). (b, d and f) Corresponding simulated STM images at constant height of isolated nanostructures (log scale intensity) for (a), (c), and (e), respectively (*V* = −1.0 V, 0 K). In all images, the brightest protrusions are assigned to iodine atoms and the less intense ones to ethyl groups.

Then, we investigated the thermal-induced reactivity of DIDEC molecules adsorbed on the Au(111) surface. Samples were heated to 378 K for 30 min. All previously described feature, including X_4_-synthon-based supramolecular rows, disappeared, leading to more compact islands (Fig. 3a and S4). A compact network of continuous backbones (highlighted in pink in Fig. 3a), along with a stepped line featuring ∼90° turns, is visible. These structures are surrounded by bright protrusions (black ellipses) and larger ones (dashed black circles). Bean-shaped features (highlighted in white) appear within the backbone, forming equilateral triangles with the brightest protrusions (side length = 0.45 ± 0.05 nm, Fig. 3a). The length of the continuous backbones was determined by statistical analysis of ten STM images with dimensions below 10 × 10 nm^2^, yielding an average length of 14 nm with a standard deviation of 4 nm. For larger STM images (over 20 × 20 nm^2^), the average length of the continuous backbones increases, reaching up to 30 nm (Fig. S5).

**Fig. 3:**
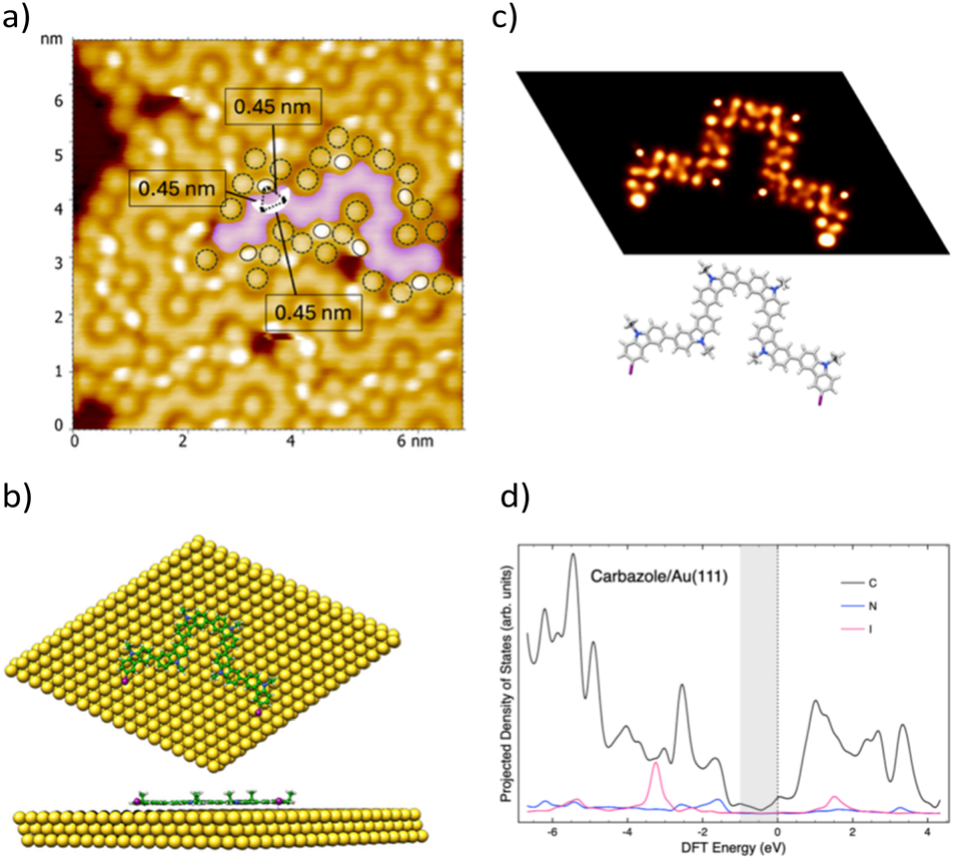
(a) STM image (6.5 × 6.5 nm^2^, *V*_s_ = 0.1 V, *I*_t_ = 40 pA, 9 K) showing nanostructures formed after deposition of 0.3 monolayers of DIDEC on Au(111) at 80 K, followed by thermal annealing at 378 K for 30 min. A compact network of continuous backbone (highlighted in pink), surrounded by bright protrusions (black ellipses) and larger ones (dashed black circles), is visible. Bean-shaped features (highlighted in white) appear within the backbone, forming equilateral triangles with the brightest protrusions (side length = 0.45 nm). (b) Top- and side-view of the optimized unit cell considered for the adsorption of a DIDEC tetramer on Au(111), (c) molecular model overlaid on the STM image shown in (a). The brightest protrusions are assigned to ethyl groups and iodine atoms. Simulated STM images of a DIDEC tetramer and the corresponding oligomer arrangement. (d) Calculated projected density of states (PDOS) of the tetramer showing that the main contribution (beyond the Au substrate) near the Fermi level arises from carbon atoms.

The continuous backbone observed in STM images recorded after thermal annealing at 378 K corresponds to the formation of polycarbazole *via* an Ullmann-type coupling between DIDEC molecules. As previously reported, the bright protrusions surrounding the continuous backbone are attributed to ethyl groups borne by nitrogen atoms, as shown in the model in Fig. 3b.

The larger single protrusions (black dashed circles in Fig. 3a) are assigned to iodine atoms adsorbed on the Au(111) surface, generated through thermally induced deiodination of DIDEC molecules.^9^ To gain further insight into the continuous backbone, a simulated STM image based on DFT calculations of a 9-ethylcarbazole tetramer adsorbed on Au(111) (Fig. 3b) was compared to the experimental STM image (Fig. 3c). In the gas phase, oligomers of 3,6-poly(9-substituted)-carbazole typically adopt a non-planar conformation.^10^ However, upon on-surface thermal polymerization of DIDEC molecules, the resulting polycarbazole backbone appears fully planar on the Au(111) surface, as evidenced in Fig. 3b. Notably, the simulated STM contrasts closely match the experimental images, reinforcing the above structural assignment. This agreement is further supported by the calculated projected density of states (PDOS), which shows that the main contribution from the adsorbate near the Fermi level arises from carbon atoms (Fig. 3d). This explains the uniform contrast observed along the continuous backbone in the experimental STM images.

The polycarbazole chains formed on the Au(111) surface are remarkably long (>20 nm) and appear free of structural defects. This high degree of order is attributed to the supramolecular pre-organization of the precursor network, which consists of columns of DIDEC molecules assembled *via* X_4_-synthons. Interestingly, this one-dimensional arrangement based on X_4_-synthons is unprecedented. In previously reported systems, supramolecular networks involving halogenated disubstituted molecules forming X_4_-synthons typically result in compact two-dimensional assemblies.^11,12^ In such cases, the four molecules forming the X_4_-synthon are arranged orthogonally to one another. In contrast, all DIDEC molecules within the observed network are aligned parallel to each other (see Fig. S6). This surprising and original organization arises from the bent geometry of the DIDEC molecule, induced by the 3,6-disubstitution pattern on the carbazole core. Attempting to form a 2D network with orthogonal X_4_-synthons using DIDEC would lead to significant steric hindrance between the ethyl groups carried by the nitrogen atoms of adjacent molecules (see Fig. S7). As a result, the system adopts an unusual configuration in which the X_4_-synthons align DIDEC molecules in nearly parallel orientations, yielding a supramolecular wire with a strictly one-dimensional character (see Fig. S8). This anisotropic pre-organization strongly favours directional, on-surface polymerization, leading to the formation of extended polycarbazole wires with high structural integrity. Furthermore, the presence of iodine atoms enables polymerization at relatively low temperatures (*ca.* 100 °C), which likely contributes to minimizing structural defects on the Au(111) surface.

In summary, we have demonstrated that selecting a bent molecular geometry—specifically, a carbazole core substituted at the 3 and 6 positions with iodine atoms—enables the formation of a one-dimensional supramolecular network based on X_4_-synthons. This 1D pre-organization serves as an effective template to guide the on-surface growth of extended, defect-free conjugated heterocyclic polymer wires. These wires are laterally decorated with substituents attached to the nitrogen atoms of the DIDEC units. Ongoing work aims to optimize the polymerization conditions to achieve longer and more functional polymer chains, with a particular focus on elucidating the role of iodine atoms in enhancing the directionality and structural control of the resulting architectures.

## Author contributions

F. C. and F. P. conceived the experiments. V. L. synthetised all molecules. J. J. and F. P. performed STM experiments. A. R. made all simulations. A. R., F. P and F. C. wrote the manuscript with inputs from all authors.

## Conflicts of interest

There are no conflicts to declare.

## Supplementary Material

NA-OLF-D5NA00708A-s001

## Data Availability

The data supporting this article have been included as part of the SI (Tables S1–S4). Additional STM images, experimental procedures and numerical simulations are detailed. See DOI: https://doi.org/10.1039/d5na00708a.
