# Supplementary material for: Halogen bonding-guided growth of heteroatom-rich polycarbazole wires on Au(111)
Source: Nanoscale Adv. 2025 Aug 20;7(19):5951–5. doi: 10.1039/d5na00708a (PMC12379804; doi:10.1039/d5na00708a)
Supplement: NA-007-D5NA00708A-s001 [file NA-007-D5NA00708A-s001.pdf]

Supplementary Information (SI) for Nanoscale Advances.
This journal is © The Royal Society of Chemistry 2025

Halogen Bonding-Guided Growth of Heteroatom-Rich Polycarbazole Wires on Au(111)

Frank Palmino,[†] *Vincent Luzet,*[†] *Judicaël Jeannoutot,*[†] *Alain Rochefort,*[‡] *and Frédéric Chérioux**[†]

[†] Université Marie et Louis PASTEUR, FEMTO-ST, CNRS, F-25000 Besançon, France

[‡] Engineering Physics Department, Polytechnique Montréal, Montréal (Québec) - H3C 3A7, Canada

Synthesis

The synthesis **3,6-diiodo-9-ethylcarbazole** was adapted from the work of K. Radula-Janik *et al.* [1] of 9-Ethylcarbazole, N-iodosuccinimide, and *para*-toluenesulfonic acid were purchased from TCI Chemicals and used as received without further purification. Ethanol was obtained from VWR International. Deuterated NMR solvents were purchased from Euriso-top. NMR spectra were recorded on a Bruker AC-300 MHz spectrometer.

3,6-diiodo-9-ethylcarbazole (DIDEC).

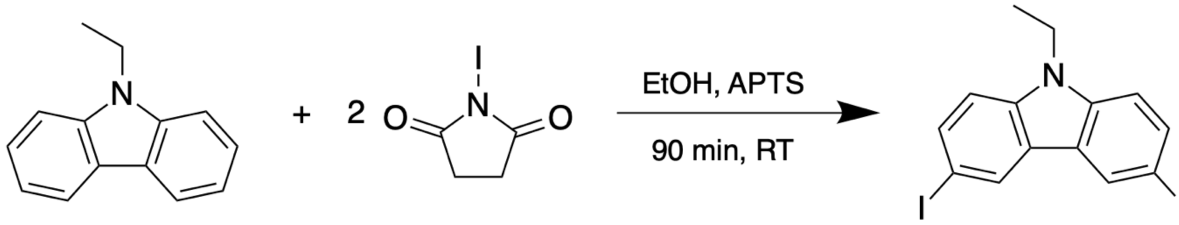

Scheme S1. Synthesis of **3,6-diiodo-9-ethylcarbazole** (DIDEC)

9-Ethylcarbazole (0.50 g, 2.56 mmol), N-iodosuccinimide (1.15 g, 5.11 mmol), and a catalytic amount of *para*-toluenesulfonic acid were dissolved in 16 mL of ethanol (Scheme 1). The resulting mixture was stirred at room temperature for 90 minutes. The crude yellow solid obtained was filtered and washed three times with 100 mL of hot ethanol to afford pure 3,6-diiodo-9-ethylcarbazole (916 mg, 2.04 mmol). The NMR spectra are detailed below (Figures S1-S2).

^{1}H NMR (300 MHz, Chloroform-d) δ (ppm) = 8.33 (d, J=1.55Hz, 2H), 7.03 (dd, J=1.55Hz, J=8.58Hz, 2H), 7.18 (d, J=8.58Hz, 2H), 4.30 (q, J=7.23Hz, 2H), 1.40 (t, J=7.23Hz, 3H). ^{13}C NMR (75 MHz, Chloroform-d) δ (ppm) = 139.0, 134.5, 129.5, 124.1, 110.7, 81.7, 37.7, 13.7.

STM Experiments.

The Au(111) surface was cleaned in a preparation chamber by sequential sputtering of argon (Ar^+) ion gun (800 eV at 3×10^{-5} mbar of Ar for 10 min) and annealed (750 K during 20 min). STM experiments were performed in a UHV chamber with a base pressure lower than 2×10^{-10} mbar using a low-temperature Scienta-infinity scanning tunneling microscope (LT-STM) with a closed-cycle cooling system. STM images were acquired in a constant current mode at 9 K. The temperature was measured using a thermocouple placed approximately 0.5 cm from the sample holder. The sample is heated with a pyrolytic boron nitride heater. DIDEC molecules were deposited from a quartz crucible at 378 K. The Au(111) surface was kept below room temperature during sublimation. The molecular coverage was determined by measuring the percentage of the area occupied by the molecules in a large set of STM images. Each image was treated with the Mountain software. The structural models are created with Blender 3D [https://www.blender.org].

Numerical simulations.

The density functional theory (DFT) calculations were performed with the Siesta package[2] (version 4.1.5) using the generalized gradient approximation (GGA) functional developed by Perdew, Burke and Ernzerhof (PBE)[3]. We have evaluated van der Waals energy through the semi-classical D3 corrections of Grimme[4]. In the PBE+D3 calculations, we used norm-conserving Trouillier-Martins pseudopotentials along with double-zeta atomic basis sets. We applied periodic boundary conditions, a mesh cutoff of 300 Ry was employed to build the real space grid. Structural relaxations were conducted with the conjugate-gradient method at Γ-point with a *k*-point mesh of 1x1x1 until the forces and the change in total energy were lower than 0.01 eV/Å and 0.00001 eV, respectively. We have considered the molecular gas phases for the monomer, dimer and tetramer, for which the geometries were fully optimized. The adsorption of poly-carbazole on Au(111) surface was modelled with an oligomer containing four carbazole units and terminated with I atoms. The oligomer was adsorbed on a three-layers gold substrate containing 972 atoms. For that system, we fully optimized the carbazole oligomer until the forces were lower than 0.02 eV/Å and 0.00001 eV for total energy, while the gold substrate geometry was kept fixed. STM simulation images were evaluated at the Tersoff–Hamann (TH) level of theory[5].

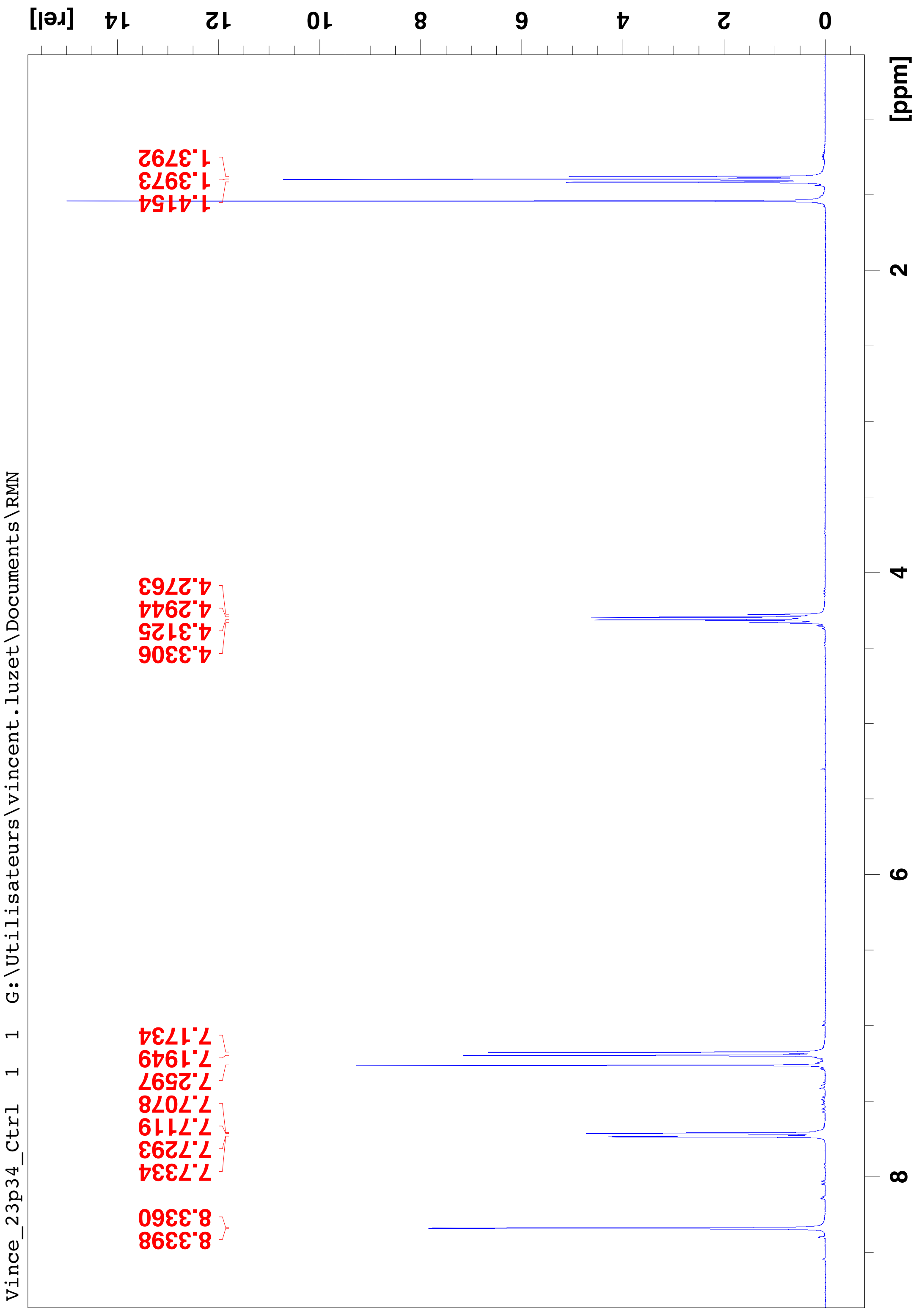

Figure S1. ^{1}H NMR spectrum of 3,6-diiodo-9-ethylcarbazole -DIDEC- molecule.

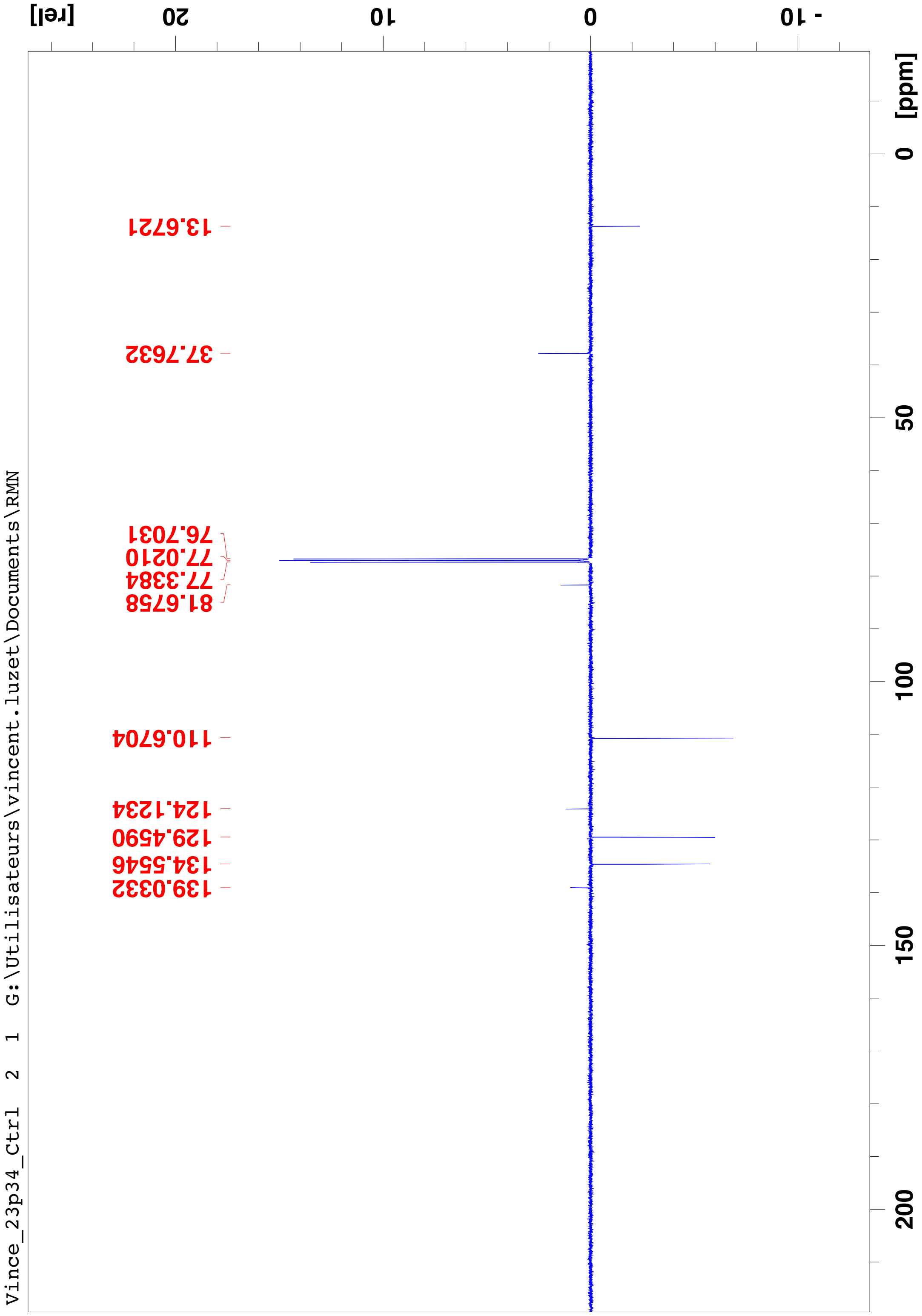

Figure S2. ^{13}C NMR spectrum of 3,6-diiodo-9-ethylcarbazole -DIDEC- molecule.

a)

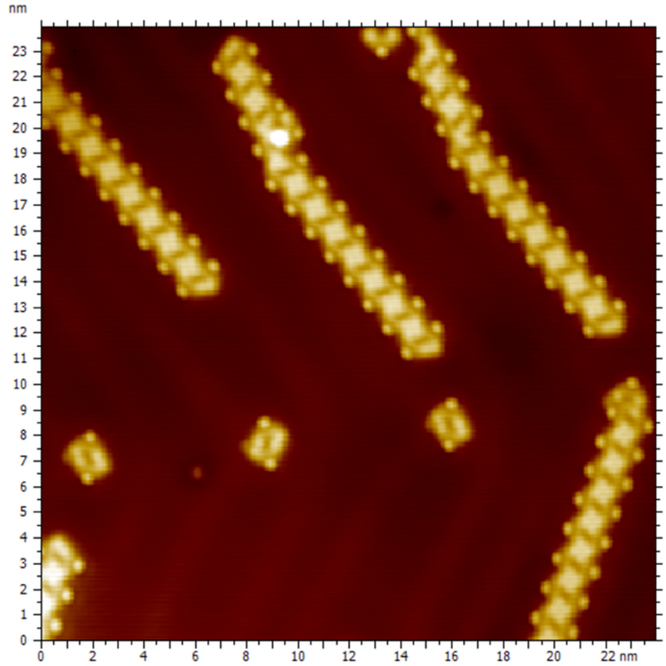

b)

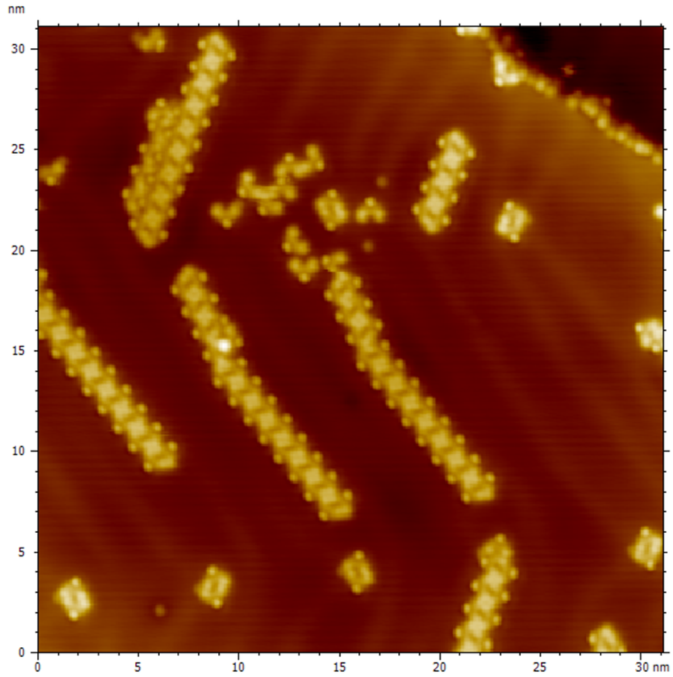

Figure S3. Two STM images (a) 24 × 24 nm^2, V_s = - 0.175 V, I_t = 1 pA and b) 31 × 31 nm^2, V_s = - 0.175 V, I_t = 1 pA) showing nanostructures formed after deposition of 0.3 monolayers of DIDEC on Au(111) at 80 K.

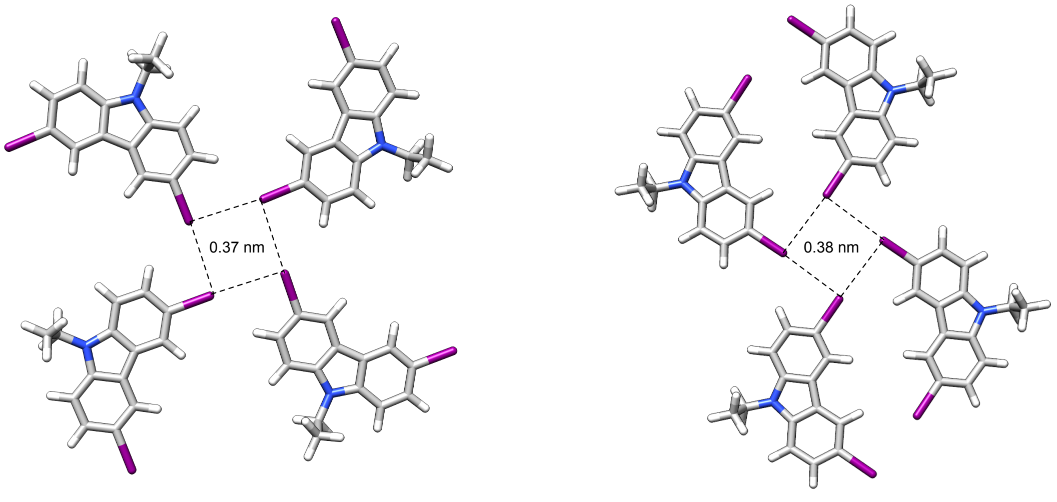

-0.32 eV/molecule   -0.48 eV/molecule

Figure S4. The cohesion energies of the DIDEC X_4-synthon compatible with the 2D structure (left) and 1D structure (right). The 1D structure is more stable.

a)

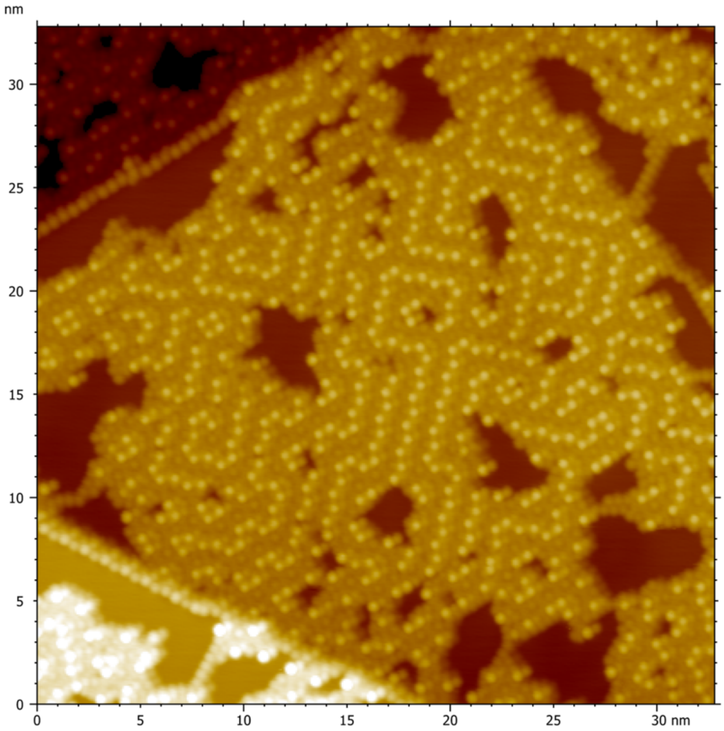

b)

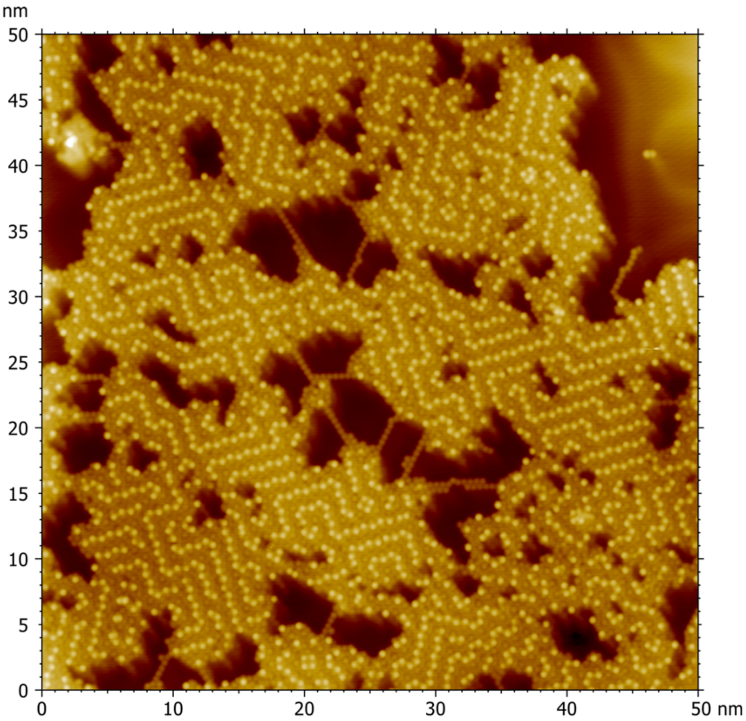

Figure S5. Two STM images (a) 33 × 33 nm^2, V_s = - 1.0 V, I_t = 10 pA and b) 50 × 50 nm^2, V_s = - 1.0 V, I_t = 10 pA) showing nanostructures formed after deposition of 0.3 monolayers of DIDEC on Au(111) at 80 K, followed by thermal annealing at 378 K for 30 min. A compact network of continuous backbones surrounded by bright protrusions is visible, along with rows of isolated protrusions on the right side of the image.

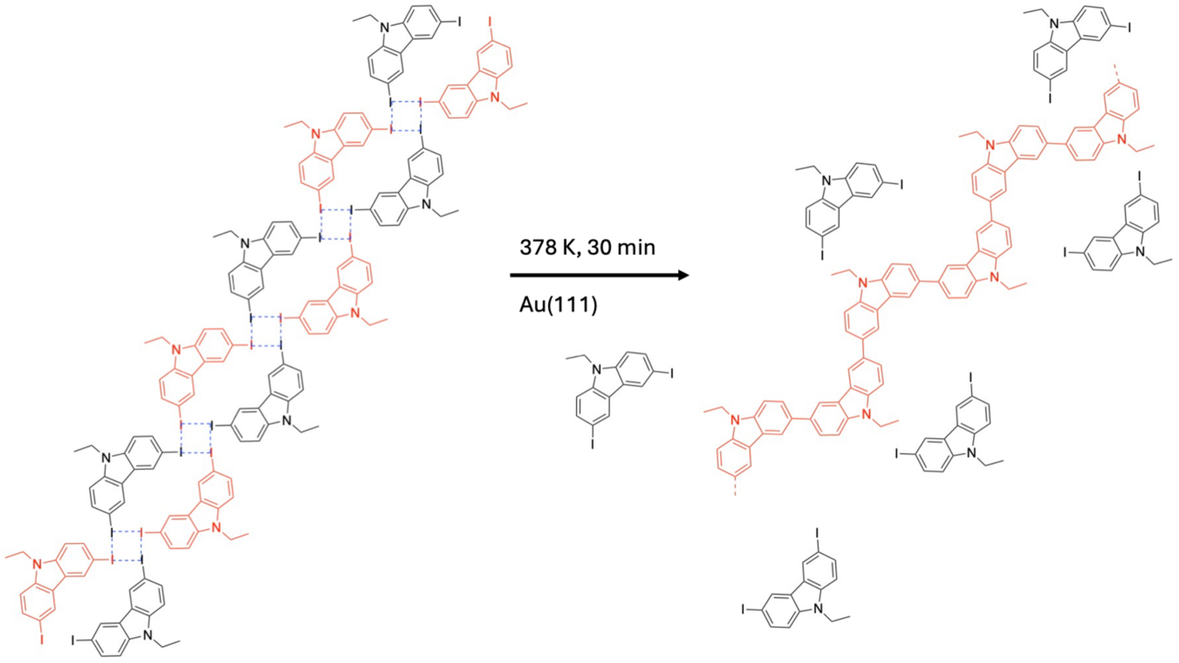

Figure S6. Thermal-induced polymerization of DIDEC molecules arrange in a X_4-synthon row (highlighted by blue-dashed square). Two DIDEC molecules are pre-organized (in red) to promote the formation of a C-C bond after deiodination. The pre-organization This anisotropic pre-organization strongly favours directional, on-surface polymerization, leading to the formation of extended polycarbazole wires with high structural integrity.

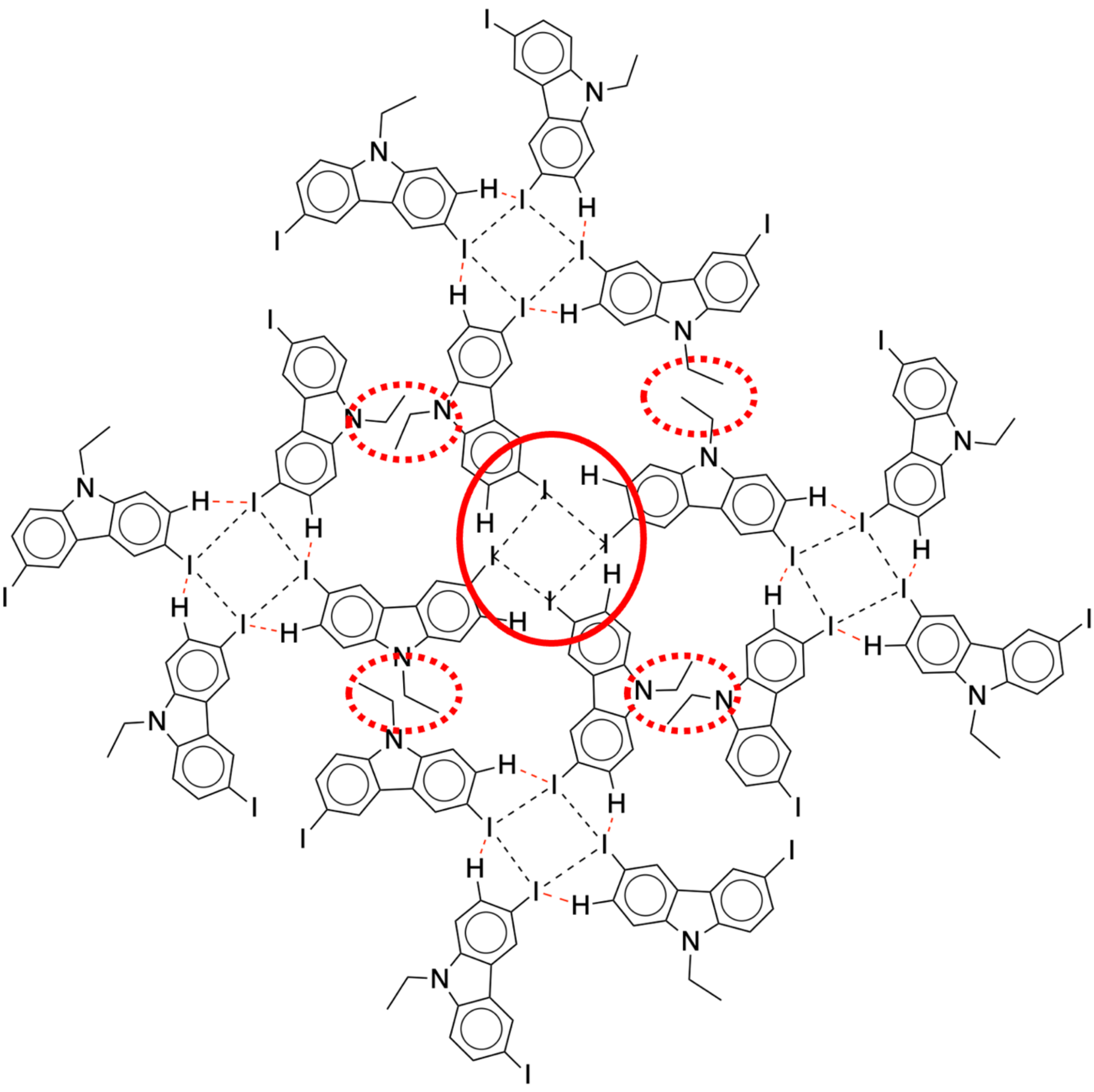

Figure S7. Hypothetical 2D arrangement of DIDEC molecules guided by X_4-synthons of halogen bonding. Steric hindrance arises from the X_4-synthons (red circles) and the ethyl groups (dashed red ellipses).

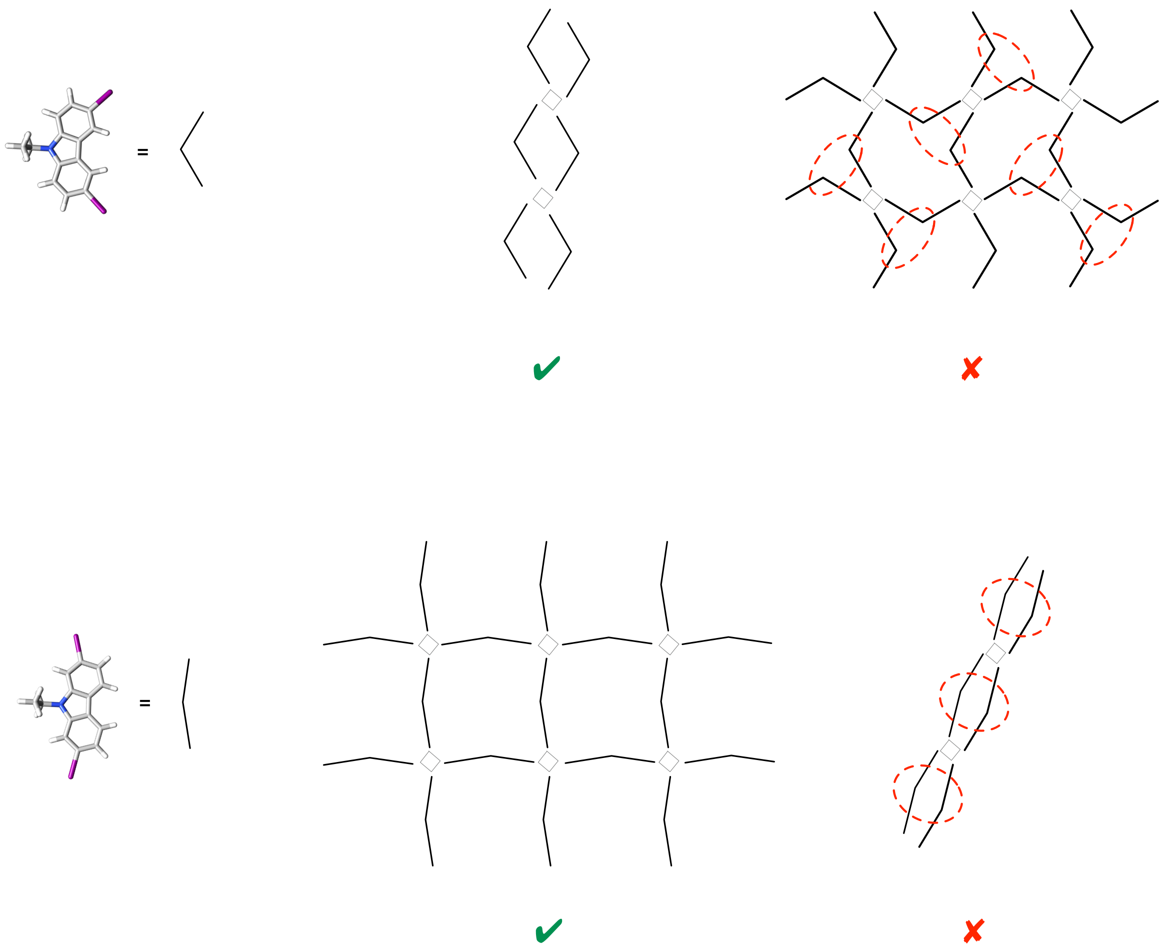

Figure S8. Comparison of supramolecular structure of 3,6-diiodo-9-ethylcarbazole (top) and 2,7-diiodo-9-ethylcarbazole (bottom). The bent configuration of 3,6-diiodo-9-ethylcarbazole promotes the formation of 1D structure.

H	35.966324	41.169254	-0.102700
H	32.218234	43.410957	0.042270
H	33.465141	45.589161	0.215802
H	38.988419	46.877798	0.250369
H	41.072626	45.475961	0.089958
H	38.705394	41.808098	-0.095253
H	35.055196	47.283495	0.626922
H	36.792494	47.690453	0.673680
H	35.721489	48.617746	-1.448040
H	36.942761	47.355550	-1.848250
H	35.192914	46.955254	-1.894506
I	41.780698	42.447145	-0.139662
I	32.925298	40.379844	-0.168276
N	36.358886	45.668094	0.266423
C	37.666685	45.117597	0.169570
C	37.571502	43.684418	0.065287
C	36.150776	43.353330	0.057995
C	35.430344	44.596558	0.156389
C	35.437844	42.129272	-0.031240
C	34.024037	42.176833	-0.032520
C	33.316040	43.415417	0.052413
C	34.014248	44.640304	0.148244
C	38.917117	45.783974	0.176880
C	40.085904	44.994570	0.087883
C	39.999867	43.571062	-0.006336
C	38.753229	42.902569	-0.017729
C	36.025225	47.114186	0.120836
C	35.966016	47.540856	-1.360256

Table S1: xyz data for single DIDEC molecules

H	35.878384	41.211843	-0.076732
H	32.173402	43.528610	0.063791
H	33.463242	45.684062	0.206732
H	39.022756	46.848283	0.225811
H	41.069612	45.393448	0.074516
H	38.602957	41.781320	-0.085892
H	35.113573	47.352327	0.649548
H	36.863392	47.705289	0.652088
H	35.785013	48.679982	-1.434099
H	36.933976	47.364733	-1.875523
H	35.166884	47.045262	-1.873442
I	41.710006	42.354576	-0.137807
I	32.800057	40.479775	-0.113312
N	36.361597	45.701370	0.248712
C	37.656253	45.121387	0.154157
C	37.527298	43.690655	0.058957

C	36.100094	43.390942	0.057916
C	35.407476	44.649774	0.148885
C	35.363165	42.180208	-0.015243
C	33.950657	42.254818	-0.010221
C	33.271291	43.510285	0.066468
C	33.992764	44.723221	0.147056
C	38.922939	45.756260	0.158447
C	40.071491	44.936252	0.074974
C	39.946635	43.515607	-0.010646
C	38.685981	42.875767	-0.018841
C	36.064568	47.155050	0.117582
C	35.980992	47.592074	-1.359289
H	36.760339	38.351466	-0.038500
H	40.463827	36.029254	0.086266
H	39.169936	33.874378	0.198672
H	33.609299	32.714069	0.163592
H	31.564956	34.174179	0.020108
H	34.036120	37.784631	-0.065007
H	37.529939	32.208574	0.589774
H	35.781708	31.845721	0.609480
H	36.856536	30.905893	-1.500381
H	35.675962	32.203779	-1.910505
H	37.436665	32.557352	-1.928673
I	30.927819	37.213911	-0.153950
I	39.839523	39.081846	-0.065186
N	36.271882	33.858090	0.225971
C	34.978196	34.440576	0.131354
C	35.108767	35.872540	0.058879
C	36.536370	36.171078	0.069682
C	37.227392	34.910628	0.146242
C	37.274769	37.382122	0.014025
C	38.687563	37.306402	0.022011
C	39.365616	36.049407	0.085898
C	38.642185	34.836480	0.149126
C	33.710868	33.806864	0.115446
C	32.563878	34.629443	0.035552
C	32.690326	36.051121	-0.027002
C	33.951511	36.689690	-0.014827
C	36.571249	32.407077	0.072588
C	36.640306	31.988865	-1.410319

Table S2: xyz data for the dimer consisting of two DIDEC molecules

H	3.756703	4.408576	-0.316630
H	7.303492	6.938464	0.047460
H	8.784620	4.918845	0.294085
H	7.723548	-0.662832	0.227427
H	5.603520	-1.990210	-0.039468
H	3.236460	1.678350	-0.328836
H	9.660858	2.740285	0.807211
H	9.332695	0.990465	0.675670
H	10.758653	1.796372	-1.292136

H	9.150685	1.206115	-1.852617
H	9.461423	2.969155	-1.723769
I	2.554737	-1.417019	-0.375239
I	4.267381	7.532418	-0.318684
N	7.688013	2.238167	0.261622
C	6.663110	1.264109	0.098800
C	5.399770	1.932930	-0.070985
C	5.670131	3.366017	-0.056579
C	7.089572	3.522025	0.124505
C	4.842644	4.511693	-0.185229
C	5.453514	5.786912	-0.143667
C	6.865404	5.931887	0.023580
C	7.700666	4.799622	0.159965
C	6.759707	-0.149590	0.105699
C	5.565780	-0.892481	-0.043244
C	4.307407	-0.233090	-0.194983
C	4.207731	1.177089	-0.212520
C	9.151615	1.970854	0.194278
C	9.667382	1.985265	-1.259437
H	0.776034	4.683912	-0.308090
H	-2.795551	2.170931	0.005459
H	-4.261291	4.197766	0.228185
H	-3.178124	9.770134	0.204300
H	-1.053669	11.094083	-0.050049
H	1.309174	7.420344	-0.334925
H	-5.138616	6.338723	0.665451
H	-4.793908	8.088652	0.738585
H	-6.174024	7.553686	-1.335969
H	-4.536658	8.146542	-1.796154
H	-4.904051	6.390898	-1.867795
I	1.979553	10.508924	-0.417491
I	0.272525	1.575043	-0.312174
N	-3.150461	6.873859	0.223782
C	-2.119684	7.841947	0.078446
C	-0.857570	7.168384	-0.082003
C	-1.135387	5.736772	-0.073231
C	-2.557841	5.586992	0.089229
C	-0.312293	4.586523	-0.191277
C	-0.932039	3.315684	-0.156839
C	-2.346175	3.173946	-0.010871
C	-3.174718	4.312265	0.113525
C	-2.214421	9.255630	0.088541
C	-1.019165	9.996834	-0.053793
C	0.235758	9.330824	-0.203179
C	0.337144	7.920845	-0.219281
C	-4.609669	7.165933	0.154386
C	-5.090576	7.324452	-1.302488
H	-3.872261	-6.612777	-0.204895
H	-7.457779	-9.095697	0.100228
H	-8.919291	-7.055895	0.286631
H	-7.796140	-1.489128	0.163064
H	-5.654508	-0.188266	-0.055382

H	-3.317806	-3.883488	-0.215465
H	-9.768144	-4.875569	0.781593
H	-9.427362	-3.127781	0.650085
H	-10.860740	-3.926354	-1.310997
H	-9.252103	-3.346303	-1.879922
H	-9.572978	-5.107618	-1.747151
I	-2.604751	-0.796986	-0.289681
I	-4.420528	-9.727556	-0.209473
N	-7.791648	-4.389359	0.240268
C	-6.752081	-3.428750	0.098606
C	-5.491611	-4.113977	-0.023905
C	-5.778821	-5.543557	-0.002835
C	-7.204474	-5.681206	0.140069
C	-4.962267	-6.700412	-0.098668
C	-5.589304	-7.967920	-0.059796
C	-7.006622	-8.094745	0.075671
C	-7.831104	-6.951162	0.178065
C	-6.834225	-2.014215	0.083281
C	-5.628227	-1.286156	-0.038550
C	-4.372983	-1.961118	-0.139852
C	-4.287619	-3.372154	-0.135626
C	-9.252684	-4.109605	0.169311
C	-9.770486	-4.121777	-1.283383
H	-0.891663	-6.895069	-0.360962
H	2.707391	-4.424052	-0.027501
H	4.146318	-6.469739	0.213690
H	2.995729	-12.026397	0.207448
H	0.858342	-13.327455	-0.062879
H	-1.460202	-9.628881	-0.378633
H	5.002553	-8.609751	0.613966
H	4.631542	-10.349700	0.763325
H	6.003174	-9.933990	-1.340611
H	4.349779	-10.505670	-1.767006
H	4.757599	-8.763907	-1.912398
I	-2.163466	-12.710641	-0.453426
I	-0.349137	-3.791893	-0.367058
N	3.003712	-9.131852	0.207123
C	1.961276	-10.086991	0.060398
C	0.708397	-9.399423	-0.112844
C	1.004391	-7.971012	-0.109510
C	2.427802	-7.837926	0.062080
C	0.196942	-6.810709	-0.238290
C	0.832267	-5.547529	-0.204608
C	2.246619	-5.422091	-0.046740
C	3.059571	-6.570444	0.087810
C	2.039472	-11.501631	0.078523
C	0.837100	-12.229772	-0.070944
C	-0.408678	-11.549903	-0.233202
C	-0.493997	-10.138912	-0.255567
C	4.457772	-9.449347	0.141648
C	4.925860	-9.677611	-1.310301

Table S3: xyz data for the X_4-synthon

C	20.600474	15.853146	12.412415
C	20.843091	15.431670	10.949833
C	21.171912	12.239312	10.742355
C	21.560382	10.872531	10.752022
C	22.938052	10.502702	10.812521
C	23.976423	11.470371	10.782884
C	22.202449	13.217349	10.795087
C	23.597090	12.844663	10.788967
C	25.232310	16.760315	10.683088
C	23.840798	16.513264	10.719373
C	23.413707	15.160089	10.783760
C	24.362907	14.080806	10.777809
C	25.755640	14.357288	10.724045
C	26.218604	15.709976	10.712031
C	33.436327	16.024778	12.382790
C	33.077217	16.308376	10.911871
C	28.152913	17.373372	10.747529
C	27.680336	16.026813	10.707994
C	28.663569	14.976560	10.637233
C	30.058715	15.225395	10.671956
C	29.545534	17.649343	10.778366
C	30.497361	16.570204	10.758190
C	29.934022	20.253622	10.779115
C	30.310290	18.886847	10.799403
C	31.701517	18.515279	10.790341
C	32.711552	19.507137	10.727794
C	32.309187	20.865038	10.706841
C	30.929549	21.276303	10.753805
C	28.031010	27.899599	12.450045
C	28.354620	27.649974	10.963644
C	28.750808	24.477832	10.711222
C	29.168535	23.125453	10.687786
C	30.551196	22.724316	10.733718
C	31.541711	23.755662	10.731575
C	33.286191	26.659430	10.708705
C	33.741028	28.016085	10.712409
C	32.745341	29.056887	10.709715
C	31.353488	28.793259	10.748684
C	29.756977	25.475044	10.783854
C	31.151523	25.120437	10.773569
C	31.900096	26.368724	10.772691
C	30.938287	27.436881	10.795060
C	40.220722	30.234112	12.770677
C	40.236263	30.160508	11.226737
C	37.066007	30.008619	10.750135
C	35.686862	29.682540	10.727706
C	35.203958	28.327472	10.698678
C	36.174698	27.276926	10.636758
C	37.999803	28.943843	10.770199

C	37.559772	27.576065	10.718685
C	41.385580	25.737866	10.742816
C	41.209826	27.141814	10.784345
C	39.885405	27.640656	10.782678
C	38.757914	26.747950	10.729473
C	38.962552	25.344591	10.670811
C	40.286250	24.804331	10.705578
C	40.419210	17.598753	12.464827
C	40.563041	17.934416	10.967930
C	41.850375	22.791264	10.658306
C	40.524917	23.326097	10.703595
C	39.428511	22.392855	10.726304
C	39.607163	20.987444	10.740160
C	42.058009	21.388803	10.731236
C	40.934107	20.492011	10.762907
C	44.640598	20.882183	10.704654
C	43.256743	20.566553	10.735443
C	42.824332	19.193712	10.759524
C	43.773263	18.140774	10.722311
C	45.149445	18.485106	10.715860
C	45.620733	19.847211	10.736783
C	52.310803	22.506212	12.438530
C	52.107042	22.150457	10.950691
C	48.905358	21.899284	10.742970
C	47.537067	21.537188	10.732744
C	47.081979	20.169829	10.750608
C	48.067347	19.136847	10.748777
C	50.901387	17.260760	10.760234
C	52.248306	16.815319	10.778407
C	53.348876	17.727053	10.738248
C	53.117097	19.128960	10.749667
C	49.859032	20.849108	10.772491
C	49.448356	19.467522	10.775353
C	50.664206	18.666067	10.777930
C	51.773522	19.585903	10.781461
N	22.095277	14.631729	10.793346
N	31.817481	17.099255	10.765617
N	29.627829	26.888307	10.796503
N	39.425127	28.987141	10.759966
N	41.404851	19.149785	10.751313
N	51.276549	20.920236	10.770615
H	33.886352	16.875062	10.406165
H	53.960175	19.832756	10.738763
H	21.456144	16.448095	12.789887
H	20.482777	14.963018	13.062106
H	19.681397	16.469263	12.475087
H	20.008670	14.816779	10.552967
H	20.926881	16.311507	10.275174
H	20.109079	12.515588	10.702890
H	20.786208	10.095369	10.701626
H	25.032838	11.172152	10.779325
H	25.550256	17.809406	10.619899

H	23.129797	17.348082	10.667200
H	26.455529	13.516558	10.725566
H	32.628863	15.456108	12.883200
H	33.580118	16.969442	12.943100
H	34.373536	15.430736	12.428306
H	33.050445	30.109137	10.650436
H	32.949102	15.367505	10.336357
H	27.454660	18.215240	10.790356
H	28.350069	13.927310	10.550364
H	30.766377	14.388705	10.602815
H	28.866727	20.499241	10.802104
H	33.776032	19.242693	10.664185
H	33.105508	21.616542	10.636215
H	28.844088	28.480115	12.932726
H	27.925711	26.936188	12.989439
H	27.079196	28.458526	12.539530
H	27.556909	27.059002	10.463806
H	28.448114	28.592478	10.379599
H	27.682153	24.725005	10.665491
H	28.380473	22.363689	10.625383
H	32.610255	23.516450	10.714789
H	33.992811	25.821909	10.686539
H	30.630478	29.618091	10.719219
H	40.636795	29.298849	13.196487
H	39.177987	30.342257	13.134258
H	40.814898	31.091125	13.142331
H	39.809411	31.063833	10.742163
H	41.257473	30.039336	10.806893
H	37.387480	31.058146	10.751296
H	34.977847	30.518873	10.736402
H	35.870464	26.225457	10.565834
H	42.419191	25.372761	10.746061
H	42.085124	27.807425	10.799251
H	38.082321	24.690667	10.617716
H	39.937531	18.436752	13.008844
H	41.412773	17.409796	12.917906
H	39.798564	16.690466	12.594679
H	41.023464	17.100270	10.393528
H	39.585404	18.133328	10.478342
H	42.730974	23.441339	10.600146
H	38.395807	22.759109	10.735470
H	38.737579	20.318307	10.737954
H	44.932774	21.938998	10.686619
H	43.468565	17.086242	10.693562
H	45.865868	17.655722	10.682147
H	52.819047	21.680285	12.975204
H	51.337634	22.682901	12.936605
H	52.923795	23.424694	12.528313
H	51.622632	22.969212	10.375713
H	53.074510	21.958829	10.438452
H	49.200107	22.957877	10.701639
H	46.809575	22.356501	10.690179

H	47.786622	18.076252	10.749599
H	50.061259	16.553062	10.744650
H	54.380873	17.346680	10.699254
I	23.434933	8.448435	10.981604
I	52.650491	14.738713	10.864655
Au	1.442498	0.832827	3.000000
Au	4.327494	0.832827	3.000000
Au	7.212489	0.832827	3.000000
Au	10.097485	0.832827	3.000000
Au	12.982481	0.832827	3.000000
Au	15.867476	0.832827	3.000000
Au	18.752472	0.832827	3.000000
Au	21.637468	0.832827	3.000000
Au	24.522463	0.832827	3.000000
Au	27.407459	0.832827	3.000000
Au	30.292455	0.832827	3.000000
Au	33.177450	0.832827	3.000000
Au	36.062446	0.832827	3.000000
Au	38.947442	0.832827	3.000000
Au	41.832437	0.832827	3.000000
Au	44.717433	0.832827	3.000000
Au	47.602429	0.832827	3.000000
Au	50.487424	0.832827	3.000000
Au	2.884996	3.331306	3.000000
Au	5.769991	3.331306	3.000000
Au	8.654987	3.331306	3.000000
Au	11.539983	3.331306	3.000000
Au	14.424978	3.331306	3.000000
Au	17.309974	3.331306	3.000000
Au	20.194970	3.331306	3.000000
Au	23.079965	3.331306	3.000000
Au	25.964961	3.331306	3.000000
Au	28.849957	3.331306	3.000000
Au	31.734952	3.331306	3.000000
Au	34.619948	3.331306	3.000000
Au	37.504944	3.331306	3.000000
Au	40.389939	3.331306	3.000000
Au	43.274935	3.331306	3.000000
Au	46.159931	3.331306	3.000000
Au	49.044926	3.331306	3.000000
Au	51.929922	3.331306	3.000000
Au	4.327494	5.829786	3.000000
Au	7.212489	5.829786	3.000000
Au	10.097485	5.829786	3.000000
Au	12.982481	5.829786	3.000000
Au	15.867476	5.829786	3.000000
Au	18.752472	5.829786	3.000000
Au	21.637468	5.829786	3.000000
Au	24.522463	5.829786	3.000000
Au	27.407459	5.829786	3.000000
Au	30.292455	5.829786	3.000000
Au	33.177450	5.829786	3.000000

Au	36.062446	5.829786	3.000000
Au	38.947442	5.829786	3.000000
Au	41.832437	5.829786	3.000000
Au	44.717433	5.829786	3.000000
Au	47.602429	5.829786	3.000000
Au	50.487424	5.829786	3.000000
Au	53.372420	5.829786	3.000000
Au	5.769991	8.328265	3.000000
Au	8.654987	8.328265	3.000000
Au	11.539983	8.328265	3.000000
Au	14.424978	8.328265	3.000000
Au	17.309974	8.328265	3.000000
Au	20.194970	8.328265	3.000000
Au	23.079965	8.328265	3.000000
Au	25.964961	8.328265	3.000000
Au	28.849957	8.328265	3.000000
Au	31.734952	8.328265	3.000000
Au	34.619948	8.328265	3.000000
Au	37.504944	8.328265	3.000000
Au	40.389939	8.328265	3.000000
Au	43.274935	8.328265	3.000000
Au	46.159931	8.328265	3.000000
Au	49.044926	8.328265	3.000000
Au	51.929922	8.328265	3.000000
Au	54.814918	8.328265	3.000000
Au	7.212489	10.826745	3.000000
Au	10.097485	10.826745	3.000000
Au	12.982481	10.826745	3.000000
Au	15.867476	10.826745	3.000000
Au	18.752472	10.826745	3.000000
Au	21.637468	10.826745	3.000000
Au	24.522463	10.826745	3.000000
Au	27.407459	10.826745	3.000000
Au	30.292455	10.826745	3.000000
Au	33.177450	10.826745	3.000000
Au	36.062446	10.826745	3.000000
Au	38.947442	10.826745	3.000000
Au	41.832437	10.826745	3.000000
Au	44.717433	10.826745	3.000000
Au	47.602429	10.826745	3.000000
Au	50.487424	10.826745	3.000000
Au	53.372420	10.826745	3.000000
Au	56.257416	10.826745	3.000000
Au	8.654987	13.325224	3.000000
Au	11.539983	13.325224	3.000000
Au	14.424978	13.325224	3.000000
Au	17.309974	13.325224	3.000000
Au	20.194970	13.325224	3.000000
Au	23.079965	13.325224	3.000000
Au	25.964961	13.325224	3.000000
Au	28.849957	13.325224	3.000000
Au	31.734952	13.325224	3.000000

Au	34.619948	13.325224	3.000000
Au	37.504944	13.325224	3.000000
Au	40.389939	13.325224	3.000000
Au	43.274935	13.325224	3.000000
Au	46.159931	13.325224	3.000000
Au	49.044926	13.325224	3.000000
Au	51.929922	13.325224	3.000000
Au	54.814918	13.325224	3.000000
Au	57.699913	13.325224	3.000000
Au	10.097485	15.823704	3.000000
Au	12.982481	15.823704	3.000000
Au	15.867476	15.823704	3.000000
Au	18.752472	15.823704	3.000000
Au	21.637468	15.823704	3.000000
Au	24.522463	15.823704	3.000000
Au	27.407459	15.823704	3.000000
Au	30.292455	15.823704	3.000000
Au	33.177450	15.823704	3.000000
Au	36.062446	15.823704	3.000000
Au	38.947442	15.823704	3.000000
Au	41.832437	15.823704	3.000000
Au	44.717433	15.823704	3.000000
Au	47.602429	15.823704	3.000000
Au	50.487424	15.823704	3.000000
Au	53.372420	15.823704	3.000000
Au	56.257416	15.823704	3.000000
Au	59.142411	15.823704	3.000000
Au	11.539983	18.322183	3.000000
Au	14.424978	18.322183	3.000000
Au	17.309974	18.322183	3.000000
Au	20.194970	18.322183	3.000000
Au	23.079965	18.322183	3.000000
Au	25.964961	18.322183	3.000000
Au	28.849957	18.322183	3.000000
Au	31.734952	18.322183	3.000000
Au	34.619948	18.322183	3.000000
Au	37.504944	18.322183	3.000000
Au	40.389939	18.322183	3.000000
Au	43.274935	18.322183	3.000000
Au	46.159931	18.322183	3.000000
Au	49.044926	18.322183	3.000000
Au	51.929922	18.322183	3.000000
Au	54.814918	18.322183	3.000000
Au	57.699913	18.322183	3.000000
Au	60.584909	18.322183	3.000000
Au	12.982481	20.820663	3.000000
Au	15.867476	20.820663	3.000000
Au	18.752472	20.820663	3.000000
Au	21.637468	20.820663	3.000000
Au	24.522463	20.820663	3.000000
Au	27.407459	20.820663	3.000000
Au	30.292455	20.820663	3.000000

Au	33.177450	20.820663	3.000000
Au	36.062446	20.820663	3.000000
Au	38.947442	20.820663	3.000000
Au	41.832437	20.820663	3.000000
Au	44.717433	20.820663	3.000000
Au	47.602429	20.820663	3.000000
Au	50.487424	20.820663	3.000000
Au	53.372420	20.820663	3.000000
Au	56.257416	20.820663	3.000000
Au	59.142411	20.820663	3.000000
Au	62.027407	20.820663	3.000000
Au	14.424978	23.319142	3.000000
Au	17.309974	23.319142	3.000000
Au	20.194970	23.319142	3.000000
Au	23.079965	23.319142	3.000000
Au	25.964961	23.319142	3.000000
Au	28.849957	23.319142	3.000000
Au	31.734952	23.319142	3.000000
Au	34.619948	23.319142	3.000000
Au	37.504944	23.319142	3.000000
Au	40.389939	23.319142	3.000000
Au	43.274935	23.319142	3.000000
Au	46.159931	23.319142	3.000000
Au	49.044926	23.319142	3.000000
Au	51.929922	23.319142	3.000000
Au	54.814918	23.319142	3.000000
Au	57.699913	23.319142	3.000000
Au	60.584909	23.319142	3.000000
Au	63.469905	23.319142	3.000000
Au	15.867476	25.817622	3.000000
Au	18.752472	25.817622	3.000000
Au	21.637468	25.817622	3.000000
Au	24.522463	25.817622	3.000000
Au	27.407459	25.817622	3.000000
Au	30.292455	25.817622	3.000000
Au	33.177450	25.817622	3.000000
Au	36.062446	25.817622	3.000000
Au	38.947442	25.817622	3.000000
Au	41.832437	25.817622	3.000000
Au	44.717433	25.817622	3.000000
Au	47.602429	25.817622	3.000000
Au	50.487424	25.817622	3.000000
Au	53.372420	25.817622	3.000000
Au	56.257416	25.817622	3.000000
Au	59.142411	25.817622	3.000000
Au	62.027407	25.817622	3.000000
Au	64.912403	25.817622	3.000000
Au	17.309974	28.316101	3.000000
Au	20.194970	28.316101	3.000000
Au	23.079965	28.316101	3.000000
Au	25.964961	28.316101	3.000000
Au	28.849957	28.316101	3.000000

Au	31.734952	28.316101	3.000000
Au	34.619948	28.316101	3.000000
Au	37.504944	28.316101	3.000000
Au	40.389939	28.316101	3.000000
Au	43.274935	28.316101	3.000000
Au	46.159931	28.316101	3.000000
Au	49.044926	28.316101	3.000000
Au	51.929922	28.316101	3.000000
Au	54.814918	28.316101	3.000000
Au	57.699913	28.316101	3.000000
Au	60.584909	28.316101	3.000000
Au	63.469905	28.316101	3.000000
Au	66.354900	28.316101	3.000000
Au	18.752472	30.814581	3.000000
Au	21.637468	30.814581	3.000000
Au	24.522463	30.814581	3.000000
Au	27.407459	30.814581	3.000000
Au	30.292455	30.814581	3.000000
Au	33.177450	30.814581	3.000000
Au	36.062446	30.814581	3.000000
Au	38.947442	30.814581	3.000000
Au	41.832437	30.814581	3.000000
Au	44.717433	30.814581	3.000000
Au	47.602429	30.814581	3.000000
Au	50.487424	30.814581	3.000000
Au	53.372420	30.814581	3.000000
Au	56.257416	30.814581	3.000000
Au	59.142411	30.814581	3.000000
Au	62.027407	30.814581	3.000000
Au	64.912403	30.814581	3.000000
Au	67.797398	30.814581	3.000000
Au	20.194970	33.313061	3.000000
Au	23.079965	33.313061	3.000000
Au	25.964961	33.313061	3.000000
Au	28.849957	33.313061	3.000000
Au	31.734952	33.313061	3.000000
Au	34.619948	33.313061	3.000000
Au	37.504944	33.313061	3.000000
Au	40.389939	33.313061	3.000000
Au	43.274935	33.313061	3.000000
Au	46.159931	33.313061	3.000000
Au	49.044926	33.313061	3.000000
Au	51.929922	33.313061	3.000000
Au	54.814918	33.313061	3.000000
Au	57.699913	33.313061	3.000000
Au	60.584909	33.313061	3.000000
Au	63.469905	33.313061	3.000000
Au	66.354900	33.313061	3.000000
Au	69.239896	33.313061	3.000000
Au	21.637468	35.811540	3.000000
Au	24.522463	35.811540	3.000000
Au	27.407459	35.811540	3.000000

Au	30.292455	35.811540	3.000000
Au	33.177450	35.811540	3.000000
Au	36.062446	35.811540	3.000000
Au	38.947442	35.811540	3.000000
Au	41.832437	35.811540	3.000000
Au	44.717433	35.811540	3.000000
Au	47.602429	35.811540	3.000000
Au	50.487424	35.811540	3.000000
Au	53.372420	35.811540	3.000000
Au	56.257416	35.811540	3.000000
Au	59.142411	35.811540	3.000000
Au	62.027407	35.811540	3.000000
Au	64.912403	35.811540	3.000000
Au	67.797398	35.811540	3.000000
Au	70.682394	35.811540	3.000000
Au	23.079965	38.310020	3.000000
Au	25.964961	38.310020	3.000000
Au	28.849957	38.310020	3.000000
Au	31.734952	38.310020	3.000000
Au	34.619948	38.310020	3.000000
Au	37.504944	38.310020	3.000000
Au	40.389939	38.310020	3.000000
Au	43.274935	38.310020	3.000000
Au	46.159931	38.310020	3.000000
Au	49.044926	38.310020	3.000000
Au	51.929922	38.310020	3.000000
Au	54.814918	38.310020	3.000000
Au	57.699913	38.310020	3.000000
Au	60.584909	38.310020	3.000000
Au	63.469905	38.310020	3.000000
Au	66.354900	38.310020	3.000000
Au	69.239896	38.310020	3.000000
Au	72.124892	38.310020	3.000000
Au	24.522463	40.808499	3.000000
Au	27.407459	40.808499	3.000000
Au	30.292455	40.808499	3.000000
Au	33.177450	40.808499	3.000000
Au	36.062446	40.808499	3.000000
Au	38.947442	40.808499	3.000000
Au	41.832437	40.808499	3.000000
Au	44.717433	40.808499	3.000000
Au	47.602429	40.808499	3.000000
Au	50.487424	40.808499	3.000000
Au	53.372420	40.808499	3.000000
Au	56.257416	40.808499	3.000000
Au	59.142411	40.808499	3.000000
Au	62.027407	40.808499	3.000000
Au	64.912403	40.808499	3.000000
Au	67.797398	40.808499	3.000000
Au	70.682394	40.808499	3.000000
Au	73.567390	40.808499	3.000000
Au	25.964961	43.306979	3.000000

Au	28.849957	43.306979	3.000000
Au	31.734952	43.306979	3.000000
Au	34.619948	43.306979	3.000000
Au	37.504944	43.306979	3.000000
Au	40.389939	43.306979	3.000000
Au	43.274935	43.306979	3.000000
Au	46.159931	43.306979	3.000000
Au	49.044926	43.306979	3.000000
Au	51.929922	43.306979	3.000000
Au	54.814918	43.306979	3.000000
Au	57.699913	43.306979	3.000000
Au	60.584909	43.306979	3.000000
Au	63.469905	43.306979	3.000000
Au	66.354900	43.306979	3.000000
Au	69.239896	43.306979	3.000000
Au	72.124892	43.306979	3.000000
Au	75.009887	43.306979	3.000000
Au	0.000000	1.665653	5.355589
Au	2.884996	1.665653	5.355589
Au	5.769991	1.665653	5.355589
Au	8.654987	1.665653	5.355589
Au	11.539983	1.665653	5.355589
Au	14.424978	1.665653	5.355589
Au	17.309974	1.665653	5.355589
Au	20.194970	1.665653	5.355589
Au	23.079965	1.665653	5.355589
Au	25.964961	1.665653	5.355589
Au	28.849957	1.665653	5.355589
Au	31.734952	1.665653	5.355589
Au	34.619948	1.665653	5.355589
Au	37.504944	1.665653	5.355589
Au	40.389939	1.665653	5.355589
Au	43.274935	1.665653	5.355589
Au	46.159931	1.665653	5.355589
Au	49.044926	1.665653	5.355589
Au	1.442498	4.164133	5.355589
Au	4.327494	4.164133	5.355589
Au	7.212489	4.164133	5.355589
Au	10.097485	4.164133	5.355589
Au	12.982481	4.164133	5.355589
Au	15.867476	4.164133	5.355589
Au	18.752472	4.164133	5.355589
Au	21.637468	4.164133	5.355589
Au	24.522463	4.164133	5.355589
Au	27.407459	4.164133	5.355589
Au	30.292455	4.164133	5.355589
Au	33.177450	4.164133	5.355589
Au	36.062446	4.164133	5.355589
Au	38.947442	4.164133	5.355589
Au	41.832437	4.164133	5.355589
Au	44.717433	4.164133	5.355589
Au	47.602429	4.164133	5.355589

Au	50.487424	4.164133	5.355589
Au	2.884996	6.662612	5.355589
Au	5.769991	6.662612	5.355589
Au	8.654987	6.662612	5.355589
Au	11.539983	6.662612	5.355589
Au	14.424978	6.662612	5.355589
Au	17.309974	6.662612	5.355589
Au	20.194970	6.662612	5.355589
Au	23.079965	6.662612	5.355589
Au	25.964961	6.662612	5.355589
Au	28.849957	6.662612	5.355589
Au	31.734952	6.662612	5.355589
Au	34.619948	6.662612	5.355589
Au	37.504944	6.662612	5.355589
Au	40.389939	6.662612	5.355589
Au	43.274935	6.662612	5.355589
Au	46.159931	6.662612	5.355589
Au	49.044926	6.662612	5.355589
Au	51.929922	6.662612	5.355589
Au	4.327494	9.161092	5.355589
Au	7.212489	9.161092	5.355589
Au	10.097485	9.161092	5.355589
Au	12.982481	9.161092	5.355589
Au	15.867476	9.161092	5.355589
Au	18.752472	9.161092	5.355589
Au	21.637468	9.161092	5.355589
Au	24.522463	9.161092	5.355589
Au	27.407459	9.161092	5.355589
Au	30.292455	9.161092	5.355589
Au	33.177450	9.161092	5.355589
Au	36.062446	9.161092	5.355589
Au	38.947442	9.161092	5.355589
Au	41.832437	9.161092	5.355589
Au	44.717433	9.161092	5.355589
Au	47.602429	9.161092	5.355589
Au	50.487424	9.161092	5.355589
Au	53.372420	9.161092	5.355589
Au	5.769991	11.659571	5.355589
Au	8.654987	11.659571	5.355589
Au	11.539983	11.659571	5.355589
Au	14.424978	11.659571	5.355589
Au	17.309974	11.659571	5.355589
Au	20.194970	11.659571	5.355589
Au	23.079965	11.659571	5.355589
Au	25.964961	11.659571	5.355589
Au	28.849957	11.659571	5.355589
Au	31.734952	11.659571	5.355589
Au	34.619948	11.659571	5.355589
Au	37.504944	11.659571	5.355589
Au	40.389939	11.659571	5.355589
Au	43.274935	11.659571	5.355589
Au	46.159931	11.659571	5.355589

Au	49.044926	11.659571	5.355589
Au	51.929922	11.659571	5.355589
Au	54.814918	11.659571	5.355589
Au	7.212489	14.158051	5.355589
Au	10.097485	14.158051	5.355589
Au	12.982481	14.158051	5.355589
Au	15.867476	14.158051	5.355589
Au	18.752472	14.158051	5.355589
Au	21.637468	14.158051	5.355589
Au	24.522463	14.158051	5.355589
Au	27.407459	14.158051	5.355589
Au	30.292455	14.158051	5.355589
Au	33.177450	14.158051	5.355589
Au	36.062446	14.158051	5.355589
Au	38.947442	14.158051	5.355589
Au	41.832437	14.158051	5.355589
Au	44.717433	14.158051	5.355589
Au	47.602429	14.158051	5.355589
Au	50.487424	14.158051	5.355589
Au	53.372420	14.158051	5.355589
Au	56.257416	14.158051	5.355589
Au	8.654987	16.656530	5.355589
Au	11.539983	16.656530	5.355589
Au	14.424978	16.656530	5.355589
Au	17.309974	16.656530	5.355589
Au	20.194970	16.656530	5.355589
Au	23.079965	16.656530	5.355589
Au	25.964961	16.656530	5.355589
Au	28.849957	16.656530	5.355589
Au	31.734952	16.656530	5.355589
Au	34.619948	16.656530	5.355589
Au	37.504944	16.656530	5.355589
Au	40.389939	16.656530	5.355589
Au	43.274935	16.656530	5.355589
Au	46.159931	16.656530	5.355589
Au	49.044926	16.656530	5.355589
Au	51.929922	16.656530	5.355589
Au	54.814918	16.656530	5.355589
Au	57.699913	16.656530	5.355589
Au	10.097485	19.155010	5.355589
Au	12.982481	19.155010	5.355589
Au	15.867476	19.155010	5.355589
Au	18.752472	19.155010	5.355589
Au	21.637468	19.155010	5.355589
Au	24.522463	19.155010	5.355589
Au	27.407459	19.155010	5.355589
Au	30.292455	19.155010	5.355589
Au	33.177450	19.155010	5.355589
Au	36.062446	19.155010	5.355589
Au	38.947442	19.155010	5.355589
Au	41.832437	19.155010	5.355589
Au	44.717433	19.155010	5.355589

Au	47.602429	19.155010	5.355589
Au	50.487424	19.155010	5.355589
Au	53.372420	19.155010	5.355589
Au	56.257416	19.155010	5.355589
Au	59.142411	19.155010	5.355589
Au	11.539983	21.653489	5.355589
Au	14.424978	21.653489	5.355589
Au	17.309974	21.653489	5.355589
Au	20.194970	21.653489	5.355589
Au	23.079965	21.653489	5.355589
Au	25.964961	21.653489	5.355589
Au	28.849957	21.653489	5.355589
Au	31.734952	21.653489	5.355589
Au	34.619948	21.653489	5.355589
Au	37.504944	21.653489	5.355589
Au	40.389939	21.653489	5.355589
Au	43.274935	21.653489	5.355589
Au	46.159931	21.653489	5.355589
Au	49.044926	21.653489	5.355589
Au	51.929922	21.653489	5.355589
Au	54.814918	21.653489	5.355589
Au	57.699913	21.653489	5.355589
Au	60.584909	21.653489	5.355589
Au	12.982481	24.151969	5.355589
Au	15.867476	24.151969	5.355589
Au	18.752472	24.151969	5.355589
Au	21.637468	24.151969	5.355589
Au	24.522463	24.151969	5.355589
Au	27.407459	24.151969	5.355589
Au	30.292455	24.151969	5.355589
Au	33.177450	24.151969	5.355589
Au	36.062446	24.151969	5.355589
Au	38.947442	24.151969	5.355589
Au	41.832437	24.151969	5.355589
Au	44.717433	24.151969	5.355589
Au	47.602429	24.151969	5.355589
Au	50.487424	24.151969	5.355589
Au	53.372420	24.151969	5.355589
Au	56.257416	24.151969	5.355589
Au	59.142411	24.151969	5.355589
Au	62.027407	24.151969	5.355589
Au	14.424978	26.650448	5.355589
Au	17.309974	26.650448	5.355589
Au	20.194970	26.650448	5.355589
Au	23.079965	26.650448	5.355589
Au	25.964961	26.650448	5.355589
Au	28.849957	26.650448	5.355589
Au	31.734952	26.650448	5.355589
Au	34.619948	26.650448	5.355589
Au	37.504944	26.650448	5.355589
Au	40.389939	26.650448	5.355589
Au	43.274935	26.650448	5.355589

Au	46.159931	26.650448	5.355589
Au	49.044926	26.650448	5.355589
Au	51.929922	26.650448	5.355589
Au	54.814918	26.650448	5.355589
Au	57.699913	26.650448	5.355589
Au	60.584909	26.650448	5.355589
Au	63.469905	26.650448	5.355589
Au	15.867476	29.148928	5.355589
Au	18.752472	29.148928	5.355589
Au	21.637468	29.148928	5.355589
Au	24.522463	29.148928	5.355589
Au	27.407459	29.148928	5.355589
Au	30.292455	29.148928	5.355589
Au	33.177450	29.148928	5.355589
Au	36.062446	29.148928	5.355589
Au	38.947442	29.148928	5.355589
Au	41.832437	29.148928	5.355589
Au	44.717433	29.148928	5.355589
Au	47.602429	29.148928	5.355589
Au	50.487424	29.148928	5.355589
Au	53.372420	29.148928	5.355589
Au	56.257416	29.148928	5.355589
Au	59.142411	29.148928	5.355589
Au	62.027407	29.148928	5.355589
Au	64.912403	29.148928	5.355589
Au	17.309974	31.647407	5.355589
Au	20.194970	31.647407	5.355589
Au	23.079965	31.647407	5.355589
Au	25.964961	31.647407	5.355589
Au	28.849957	31.647407	5.355589
Au	31.734952	31.647407	5.355589
Au	34.619948	31.647407	5.355589
Au	37.504944	31.647407	5.355589
Au	40.389939	31.647407	5.355589
Au	43.274935	31.647407	5.355589
Au	46.159931	31.647407	5.355589
Au	49.044926	31.647407	5.355589
Au	51.929922	31.647407	5.355589
Au	54.814918	31.647407	5.355589
Au	57.699913	31.647407	5.355589
Au	60.584909	31.647407	5.355589
Au	63.469905	31.647407	5.355589
Au	66.354900	31.647407	5.355589
Au	18.752472	34.145887	5.355589
Au	21.637468	34.145887	5.355589
Au	24.522463	34.145887	5.355589
Au	27.407459	34.145887	5.355589
Au	30.292455	34.145887	5.355589
Au	33.177450	34.145887	5.355589
Au	36.062446	34.145887	5.355589
Au	38.947442	34.145887	5.355589
Au	41.832437	34.145887	5.355589

Au	44.717433	34.145887	5.355589
Au	47.602429	34.145887	5.355589
Au	50.487424	34.145887	5.355589
Au	53.372420	34.145887	5.355589
Au	56.257416	34.145887	5.355589
Au	59.142411	34.145887	5.355589
Au	62.027407	34.145887	5.355589
Au	64.912403	34.145887	5.355589
Au	67.797398	34.145887	5.355589
Au	20.194970	36.644367	5.355589
Au	23.079965	36.644367	5.355589
Au	25.964961	36.644367	5.355589
Au	28.849957	36.644367	5.355589
Au	31.734952	36.644367	5.355589
Au	34.619948	36.644367	5.355589
Au	37.504944	36.644367	5.355589
Au	40.389939	36.644367	5.355589
Au	43.274935	36.644367	5.355589
Au	46.159931	36.644367	5.355589
Au	49.044926	36.644367	5.355589
Au	51.929922	36.644367	5.355589
Au	54.814918	36.644367	5.355589
Au	57.699913	36.644367	5.355589
Au	60.584909	36.644367	5.355589
Au	63.469905	36.644367	5.355589
Au	66.354900	36.644367	5.355589
Au	69.239896	36.644367	5.355589
Au	21.637468	39.142846	5.355589
Au	24.522463	39.142846	5.355589
Au	27.407459	39.142846	5.355589
Au	30.292455	39.142846	5.355589
Au	33.177450	39.142846	5.355589
Au	36.062446	39.142846	5.355589
Au	38.947442	39.142846	5.355589
Au	41.832437	39.142846	5.355589
Au	44.717433	39.142846	5.355589
Au	47.602429	39.142846	5.355589
Au	50.487424	39.142846	5.355589
Au	53.372420	39.142846	5.355589
Au	56.257416	39.142846	5.355589
Au	59.142411	39.142846	5.355589
Au	62.027407	39.142846	5.355589
Au	64.912403	39.142846	5.355589
Au	67.797398	39.142846	5.355589
Au	70.682394	39.142846	5.355589
Au	23.079965	41.641326	5.355589
Au	25.964961	41.641326	5.355589
Au	28.849957	41.641326	5.355589
Au	31.734952	41.641326	5.355589
Au	34.619948	41.641326	5.355589
Au	37.504944	41.641326	5.355589
Au	40.389939	41.641326	5.355589

Au	43.274935	41.641326	5.355589
Au	46.159931	41.641326	5.355589
Au	49.044926	41.641326	5.355589
Au	51.929922	41.641326	5.355589
Au	54.814918	41.641326	5.355589
Au	57.699913	41.641326	5.355589
Au	60.584909	41.641326	5.355589
Au	63.469905	41.641326	5.355589
Au	66.354900	41.641326	5.355589
Au	69.239896	41.641326	5.355589
Au	72.124892	41.641326	5.355589
Au	24.522463	44.139805	5.355589
Au	27.407459	44.139805	5.355589
Au	30.292455	44.139805	5.355589
Au	33.177450	44.139805	5.355589
Au	36.062446	44.139805	5.355589
Au	38.947442	44.139805	5.355589
Au	41.832437	44.139805	5.355589
Au	44.717433	44.139805	5.355589
Au	47.602429	44.139805	5.355589
Au	50.487424	44.139805	5.355589
Au	53.372420	44.139805	5.355589
Au	56.257416	44.139805	5.355589
Au	59.142411	44.139805	5.355589
Au	62.027407	44.139805	5.355589
Au	64.912403	44.139805	5.355589
Au	67.797398	44.139805	5.355589
Au	70.682394	44.139805	5.355589
Au	73.567390	44.139805	5.355589
Au	0.000000	0.000000	7.711178
Au	2.884996	0.000000	7.711178
Au	5.769991	0.000000	7.711178
Au	8.654987	0.000000	7.711178
Au	11.539983	0.000000	7.711178
Au	14.424978	0.000000	7.711178
Au	17.309974	0.000000	7.711178
Au	20.194970	0.000000	7.711178
Au	23.079965	0.000000	7.711178
Au	25.964961	0.000000	7.711178
Au	28.849957	0.000000	7.711178
Au	31.734952	0.000000	7.711178
Au	34.619948	0.000000	7.711178
Au	37.504944	0.000000	7.711178
Au	40.389939	0.000000	7.711178
Au	43.274935	0.000000	7.711178
Au	46.159931	0.000000	7.711178
Au	49.044926	0.000000	7.711178
Au	1.442498	2.498480	7.711178
Au	4.327494	2.498480	7.711178
Au	7.212489	2.498480	7.711178
Au	10.097485	2.498480	7.711178
Au	12.982481	2.498480	7.711178

Au	15.867476	2.498480	7.711178
Au	18.752472	2.498480	7.711178
Au	21.637468	2.498480	7.711178
Au	24.522463	2.498480	7.711178
Au	27.407459	2.498480	7.711178
Au	30.292455	2.498480	7.711178
Au	33.177450	2.498480	7.711178
Au	36.062446	2.498480	7.711178
Au	38.947442	2.498480	7.711178
Au	41.832437	2.498480	7.711178
Au	44.717433	2.498480	7.711178
Au	47.602429	2.498480	7.711178
Au	50.487424	2.498480	7.711178
Au	2.884996	4.996959	7.711178
Au	5.769991	4.996959	7.711178
Au	8.654987	4.996959	7.711178
Au	11.539983	4.996959	7.711178
Au	14.424978	4.996959	7.711178
Au	17.309974	4.996959	7.711178
Au	20.194970	4.996959	7.711178
Au	23.079965	4.996959	7.711178
Au	25.964961	4.996959	7.711178
Au	28.849957	4.996959	7.711178
Au	31.734952	4.996959	7.711178
Au	34.619948	4.996959	7.711178
Au	37.504944	4.996959	7.711178
Au	40.389939	4.996959	7.711178
Au	43.274935	4.996959	7.711178
Au	46.159931	4.996959	7.711178
Au	49.044926	4.996959	7.711178
Au	51.929922	4.996959	7.711178
Au	4.327494	7.495439	7.711178
Au	7.212489	7.495439	7.711178
Au	10.097485	7.495439	7.711178
Au	12.982481	7.495439	7.711178
Au	15.867476	7.495439	7.711178
Au	18.752472	7.495439	7.711178
Au	21.637468	7.495439	7.711178
Au	24.522463	7.495439	7.711178
Au	27.407459	7.495439	7.711178
Au	30.292455	7.495439	7.711178
Au	33.177450	7.495439	7.711178
Au	36.062446	7.495439	7.711178
Au	38.947442	7.495439	7.711178
Au	41.832437	7.495439	7.711178
Au	44.717433	7.495439	7.711178
Au	47.602429	7.495439	7.711178
Au	50.487424	7.495439	7.711178
Au	53.372420	7.495439	7.711178
Au	5.769991	9.993918	7.711178
Au	8.654987	9.993918	7.711178
Au	11.539983	9.993918	7.711178

Au	14.424978	9.993918	7.711178
Au	17.309974	9.993918	7.711178
Au	20.194970	9.993918	7.711178
Au	23.079965	9.993918	7.711178
Au	25.964961	9.993918	7.711178
Au	28.849957	9.993918	7.711178
Au	31.734952	9.993918	7.711178
Au	34.619948	9.993918	7.711178
Au	37.504944	9.993918	7.711178
Au	40.389939	9.993918	7.711178
Au	43.274935	9.993918	7.711178
Au	46.159931	9.993918	7.711178
Au	49.044926	9.993918	7.711178
Au	51.929922	9.993918	7.711178
Au	54.814918	9.993918	7.711178
Au	7.212489	12.492398	7.711178
Au	10.097485	12.492398	7.711178
Au	12.982481	12.492398	7.711178
Au	15.867476	12.492398	7.711178
Au	18.752472	12.492398	7.711178
Au	21.637468	12.492398	7.711178
Au	24.522463	12.492398	7.711178
Au	27.407459	12.492398	7.711178
Au	30.292455	12.492398	7.711178
Au	33.177450	12.492398	7.711178
Au	36.062446	12.492398	7.711178
Au	38.947442	12.492398	7.711178
Au	41.832437	12.492398	7.711178
Au	44.717433	12.492398	7.711178
Au	47.602429	12.492398	7.711178
Au	50.487424	12.492398	7.711178
Au	53.372420	12.492398	7.711178
Au	56.257416	12.492398	7.711178
Au	8.654987	14.990877	7.711178
Au	11.539983	14.990877	7.711178
Au	14.424978	14.990877	7.711178
Au	17.309974	14.990877	7.711178
Au	20.194970	14.990877	7.711178
Au	23.079965	14.990877	7.711178
Au	25.964961	14.990877	7.711178
Au	28.849957	14.990877	7.711178
Au	31.734952	14.990877	7.711178
Au	34.619948	14.990877	7.711178
Au	37.504944	14.990877	7.711178
Au	40.389939	14.990877	7.711178
Au	43.274935	14.990877	7.711178
Au	46.159931	14.990877	7.711178
Au	49.044926	14.990877	7.711178
Au	51.929922	14.990877	7.711178
Au	54.814918	14.990877	7.711178
Au	57.699913	14.990877	7.711178
Au	10.097485	17.489357	7.711178

Au	12.982481	17.489357	7.711178
Au	15.867476	17.489357	7.711178
Au	18.752472	17.489357	7.711178
Au	21.637468	17.489357	7.711178
Au	24.522463	17.489357	7.711178
Au	27.407459	17.489357	7.711178
Au	30.292455	17.489357	7.711178
Au	33.177450	17.489357	7.711178
Au	36.062446	17.489357	7.711178
Au	38.947442	17.489357	7.711178
Au	41.832437	17.489357	7.711178
Au	44.717433	17.489357	7.711178
Au	47.602429	17.489357	7.711178
Au	50.487424	17.489357	7.711178
Au	53.372420	17.489357	7.711178
Au	56.257416	17.489357	7.711178
Au	59.142411	17.489357	7.711178
Au	11.539983	19.987836	7.711178
Au	14.424978	19.987836	7.711178
Au	17.309974	19.987836	7.711178
Au	20.194970	19.987836	7.711178
Au	23.079965	19.987836	7.711178
Au	25.964961	19.987836	7.711178
Au	28.849957	19.987836	7.711178
Au	31.734952	19.987836	7.711178
Au	34.619948	19.987836	7.711178
Au	37.504944	19.987836	7.711178
Au	40.389939	19.987836	7.711178
Au	43.274935	19.987836	7.711178
Au	46.159931	19.987836	7.711178
Au	49.044926	19.987836	7.711178
Au	51.929922	19.987836	7.711178
Au	54.814918	19.987836	7.711178
Au	57.699913	19.987836	7.711178
Au	60.584909	19.987836	7.711178
Au	12.982481	22.486316	7.711178
Au	15.867476	22.486316	7.711178
Au	18.752472	22.486316	7.711178
Au	21.637468	22.486316	7.711178
Au	24.522463	22.486316	7.711178
Au	27.407459	22.486316	7.711178
Au	30.292455	22.486316	7.711178
Au	33.177450	22.486316	7.711178
Au	36.062446	22.486316	7.711178
Au	38.947442	22.486316	7.711178
Au	41.832437	22.486316	7.711178
Au	44.717433	22.486316	7.711178
Au	47.602429	22.486316	7.711178
Au	50.487424	22.486316	7.711178
Au	53.372420	22.486316	7.711178
Au	56.257416	22.486316	7.711178
Au	59.142411	22.486316	7.711178

Au	62.027407	22.486316	7.711178
Au	14.424978	24.984795	7.711178
Au	17.309974	24.984795	7.711178
Au	20.194970	24.984795	7.711178
Au	23.079965	24.984795	7.711178
Au	25.964961	24.984795	7.711178
Au	28.849957	24.984795	7.711178
Au	31.734952	24.984795	7.711178
Au	34.619948	24.984795	7.711178
Au	37.504944	24.984795	7.711178
Au	40.389939	24.984795	7.711178
Au	43.274935	24.984795	7.711178
Au	46.159931	24.984795	7.711178
Au	49.044926	24.984795	7.711178
Au	51.929922	24.984795	7.711178
Au	54.814918	24.984795	7.711178
Au	57.699913	24.984795	7.711178
Au	60.584909	24.984795	7.711178
Au	63.469905	24.984795	7.711178
Au	15.867476	27.483275	7.711178
Au	18.752472	27.483275	7.711178
Au	21.637468	27.483275	7.711178
Au	24.522463	27.483275	7.711178
Au	27.407459	27.483275	7.711178
Au	30.292455	27.483275	7.711178
Au	33.177450	27.483275	7.711178
Au	36.062446	27.483275	7.711178
Au	38.947442	27.483275	7.711178
Au	41.832437	27.483275	7.711178
Au	44.717433	27.483275	7.711178
Au	47.602429	27.483275	7.711178
Au	50.487424	27.483275	7.711178
Au	53.372420	27.483275	7.711178
Au	56.257416	27.483275	7.711178
Au	59.142411	27.483275	7.711178
Au	62.027407	27.483275	7.711178
Au	64.912403	27.483275	7.711178
Au	17.309974	29.981754	7.711178
Au	20.194970	29.981754	7.711178
Au	23.079965	29.981754	7.711178
Au	25.964961	29.981754	7.711178
Au	28.849957	29.981754	7.711178
Au	31.734952	29.981754	7.711178
Au	34.619948	29.981754	7.711178
Au	37.504944	29.981754	7.711178
Au	40.389939	29.981754	7.711178
Au	43.274935	29.981754	7.711178
Au	46.159931	29.981754	7.711178
Au	49.044926	29.981754	7.711178
Au	51.929922	29.981754	7.711178
Au	54.814918	29.981754	7.711178
Au	57.699913	29.981754	7.711178

Au	60.584909	29.981754	7.711178
Au	63.469905	29.981754	7.711178
Au	66.354900	29.981754	7.711178
Au	18.752472	32.480234	7.711178
Au	21.637468	32.480234	7.711178
Au	24.522463	32.480234	7.711178
Au	27.407459	32.480234	7.711178
Au	30.292455	32.480234	7.711178
Au	33.177450	32.480234	7.711178
Au	36.062446	32.480234	7.711178
Au	38.947442	32.480234	7.711178
Au	41.832437	32.480234	7.711178
Au	44.717433	32.480234	7.711178
Au	47.602429	32.480234	7.711178
Au	50.487424	32.480234	7.711178
Au	53.372420	32.480234	7.711178
Au	56.257416	32.480234	7.711178
Au	59.142411	32.480234	7.711178
Au	62.027407	32.480234	7.711178
Au	64.912403	32.480234	7.711178
Au	67.797398	32.480234	7.711178
Au	20.194970	34.978714	7.711178
Au	23.079965	34.978714	7.711178
Au	25.964961	34.978714	7.711178
Au	28.849957	34.978714	7.711178
Au	31.734952	34.978714	7.711178
Au	34.619948	34.978714	7.711178
Au	37.504944	34.978714	7.711178
Au	40.389939	34.978714	7.711178
Au	43.274935	34.978714	7.711178
Au	46.159931	34.978714	7.711178
Au	49.044926	34.978714	7.711178
Au	51.929922	34.978714	7.711178
Au	54.814918	34.978714	7.711178
Au	57.699913	34.978714	7.711178
Au	60.584909	34.978714	7.711178
Au	63.469905	34.978714	7.711178
Au	66.354900	34.978714	7.711178
Au	69.239896	34.978714	7.711178
Au	21.637468	37.477193	7.711178
Au	24.522463	37.477193	7.711178
Au	27.407459	37.477193	7.711178
Au	30.292455	37.477193	7.711178
Au	33.177450	37.477193	7.711178
Au	36.062446	37.477193	7.711178
Au	38.947442	37.477193	7.711178
Au	41.832437	37.477193	7.711178
Au	44.717433	37.477193	7.711178
Au	47.602429	37.477193	7.711178
Au	50.487424	37.477193	7.711178
Au	53.372420	37.477193	7.711178
Au	56.257416	37.477193	7.711178

Au	59.142411	37.477193	7.711178
Au	62.027407	37.477193	7.711178
Au	64.912403	37.477193	7.711178
Au	67.797398	37.477193	7.711178
Au	70.682394	37.477193	7.711178
Au	23.079965	39.975673	7.711178
Au	25.964961	39.975673	7.711178
Au	28.849957	39.975673	7.711178
Au	31.734952	39.975673	7.711178
Au	34.619948	39.975673	7.711178
Au	37.504944	39.975673	7.711178
Au	40.389939	39.975673	7.711178
Au	43.274935	39.975673	7.711178
Au	46.159931	39.975673	7.711178
Au	49.044926	39.975673	7.711178
Au	51.929922	39.975673	7.711178
Au	54.814918	39.975673	7.711178
Au	57.699913	39.975673	7.711178
Au	60.584909	39.975673	7.711178
Au	63.469905	39.975673	7.711178
Au	66.354900	39.975673	7.711178
Au	69.239896	39.975673	7.711178
Au	72.124892	39.975673	7.711178
Au	24.522463	42.474152	7.711178
Au	27.407459	42.474152	7.711178
Au	30.292455	42.474152	7.711178
Au	33.177450	42.474152	7.711178
Au	36.062446	42.474152	7.711178
Au	38.947442	42.474152	7.711178
Au	41.832437	42.474152	7.711178
Au	44.717433	42.474152	7.711178
Au	47.602429	42.474152	7.711178
Au	50.487424	42.474152	7.711178
Au	53.372420	42.474152	7.711178
Au	56.257416	42.474152	7.711178
Au	59.142411	42.474152	7.711178
Au	62.027407	42.474152	7.711178
Au	64.912403	42.474152	7.711178
Au	67.797398	42.474152	7.711178
Au	70.682394	42.474152	7.711178
Au	73.567390	42.474152	7.711178

Table S4: xyz data for oligomer adsorbed on a Au(111) surface

References

1. K. Radula-Janik, T. Kupka, K. Ejsmont, Z. Daszkiewicz, S. P. A. Sauer. *Struct. Chem.* 2016, **27**, 199.
2. J. M. Soler, E. Artacho, J. D. Gale, A. García, J. Junquera, P. Ordejón, and D. Sánchez-Portal, *J. Condens. Matter Phys.* 2002, **14**, 2745.

3. M. Ernzerhof, and G. E. Scuseria, *J. Chem. Phys.* 1999, **110**, 5029.
4. S. Grimme, *J. Comput. Chem.* 2006, **27**, 1787.
5. J. Tersoff, D.R. Hamann, *Phys. Rev. B* 1985, **31**, 805.
